# Novel Quinoline Chemosensor with Dual-Mode Fluorescence and DFT-Backed Mechanism for Mercury(II) Sensing

**DOI:** 10.1007/s10895-025-04702-3

**Published:** 2026-02-11

**Authors:** Gasser M. Khairy, Bader M. Alanazi, Yasser A. Attia, Mohamed M. Aboelnga, Randa M. Abdel Hameed

**Affiliations:** 1 Chemistry Department, Faculty of Science, Suez Canal University, Ismailia, 41522 Egypt; 2 Department of Chemistry, Faculty of Science, Cairo University, Giza, 12613 Egypt; 3 National Institute of Laser Enhanced Sciences Cairo University, Giza, 12613 Egypt; 4 Chemistry Department, Faculty of Science, Damietta University, New Damietta, 34517 Egypt; 5 Faculty of Basic Sciences, King Salman International University, Ras Sudr, South Sinai 46612 Egypt

**Keywords:** Chemosensor, Mercury detection (Hg²⁺), DFT calculations, Environmental monitoring, Selectivity and sensitivity, Water quality assessment

## Abstract

Mercury (Hg²⁺) contamination poses a significant threat to environmental and human health, necessitating the development of sensitive and selective detection methods. In this study, we report the design and application of a novel quinoline-based chemosensor, HMQC, for the detection of Hg²⁺ in aqueous environments. The probe exhibits dual fluorescence behavior, with quenching at acidic pH (pH 5) and enhancement at neutral pH (pH 7), enabling both qualitative and quantitative detection of Hg²⁺. Under optimized conditions at pH 7, HMQC demonstrated a broad linear range (4.84–50 nM), a low limit of detection (LOD) of 1.47 nM, and high selectivity toward Hg²⁺ over competing metal ions. At pH 5, the probe showed effective quenching with a linear range of 3.09–15 µM and an LOD of 0.93 µM, making it suitable for environments with higher mercury concentrations. The practical utility of HMQC was validated by detecting Hg²⁺ in spiked real water samples with recovery rates ranging from 92% to 108% and relative standard deviations (RSD) below 5%. The probe’s reversibility and reusability were confirmed through successive binding and displacement cycles with EDTA, highlighting its robustness for real-time and continuous monitoring. The adaptability of HMQC under different pH conditions aligns with international water quality standards, such as the World Health Organization (WHO) and European Union (EU) limits for mercury in drinking water (1 µg/L or 5 nM), demonstrating its suitability for environmental and industrial applications. Moreover, DFT calculations have been conducted to obtain atomistic insights into the binding complex and the quenching mechanism. This work provides a versatile and efficient platform for mercury detection, combining high sensitivity, selectivity, and robust performance in complex matrices. The dual fluorescence response of HMQC represents a novel approach in chemosensor design, offering significant potential for future advancements in environmental monitoring and public health protection. *Corresponding Authors. Gasser M. Khairy: gasser_mostafa@science.suez.edu.eg.

**Supplementary Information:**

The online version contains supplementary material available at 10.1007/s10895-025-04702-3.

## Introduction

Mercury (Hg²⁺) contamination is a pervasive environmental problem due to its toxicity, persistence, and bioaccumulation in ecosystems. As a potent neurotoxin, mercury poses severe risks to human health and the environment, with impacts ranging from developmental disorders to organ damage and mortality in extreme cases. Once released, mercury transforms into highly toxic methylmercury, which bioaccumulates in aquatic food chains [1–4]. International regulatory bodies, including the World Health Organization (WHO) and United States Environmental Protection Agency (EPA), have established stringent limits for mercury in drinking water at 1 µg/L (5 nM) and 2 µg/L (10 nM), respectively, emphasizing the need for reliable detection methods [5, 6]. Conventional mercury detection techniques, such as atomic absorption spectroscopy (AAS) [7], inductively coupled plasma mass spectrometry (ICP-MS) [8], and cold vapor atomic fluorescence spectrometry (CV-AFS) [9], offer high sensitivity but are costly, time-consuming, and require sophisticated instrumentation [10, 11]. These limitations have spurred interest in the development of fluorescence-based chemosensors for mercury detection. Recent advances in optical sensing technologies have produced a wide variety of fluorescence- and colorimetry-based platforms for Hg²⁺ detection. These include turn-on/turn-off probes, ratiometric systems, ICT- and PET-responsive fluorophores, AIE-active structures, and hybrid organic–inorganic sensing frameworks [12–14]. A series of comprehensive reviews has also highlighted major progress in heteroatom-containing organic sensors, including quinoline, thiazole, thiourea, hydrazide, and pyrazolate scaffolds, which continue to play a central role in selective heavy-metal detection [15–21]. These optical approaches offer rapid response, high sensitivity, and structural tunability, and they form the scientific foundation upon which the present work is built. Such sensors provide a simpler, faster, and cost-effective alternative, offering high selectivity, sensitivity, and real-time monitoring capabilities [22, 23]. Recent advances in chemosensor design have led to the development of various fluorescence-based probes utilizing different mechanisms, including fluorescence quenching and enhancement [10]. Common fluorophores such as rhodamine [24], coumarin [25], and BODIPY [26] have been extensively studied. However, these probes often face challenges like limited solubility, photobleaching, and interference from competing metal ions in complex matrices [27, 28]. Addressing these challenges, pH-responsive and water-soluble chemosensors have emerged as promising candidates for real-world applications [29, 30]. In this study, we introduce a novel quinoline-based chemosensor, methyl 4-hydroxy-2-oxo-1,2-tetrahydroquinoline-3-carboxylate (HMQC), designed for the sensitive and selective detection of Hg²⁺ in aqueous environments. HMQC leverages dual fluorescence behavior, exhibiting quenching at acidic pH (pH 5) and enhancement at neutral pH (pH 7). This dual behavior allows its application across diverse environmental conditions, enhancing its versatility as a detection platform. Under optimized conditions, HMQC demonstrated a broad dynamic range and a limit of detection as low as 1.47 nM at pH 7, making it compliant with international standards for drinking water safety [5]. Moreover, the probe displayed exceptional selectivity toward Hg²⁺, even in the presence of common competing metal ions, and was successfully applied to detect Hg²⁺ in real water samples with recovery rates exceeding 92%. The quenching mechanism has been clearly explained by applying DFT calculations on the possible geometrical structure for the Hg(II)-sensor complex. This type of analysis enhanced our understanding regarding the impact of metal ligation on the electronic distribution of the sensor. This work underscores the significance of HMQC as a sensitive, selective, and practical tool for mercury detection. By addressing limitations of existing fluorescent probes and aligning with international regulatory standards, HMQC establishes itself as a valuable chemosensor for environmental monitoring and public health protection.

## Experimental

### Materials and Chemicals

**Scheme 1:**
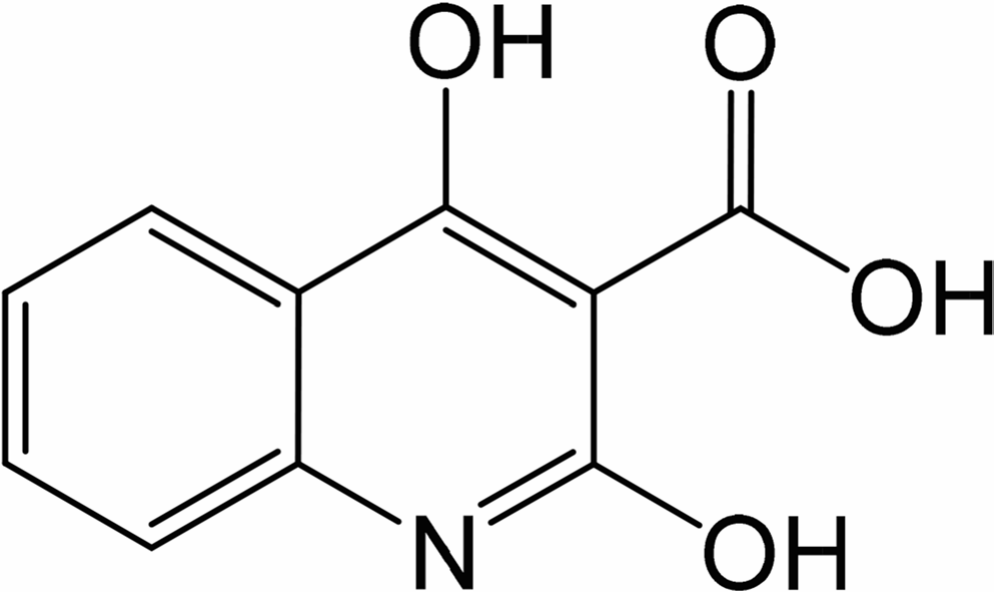
The chemical structure of sensing reagent under study (HMQC)

Methyl 4-hydroxy-2-oxo-1,2-tetrahydroquinoline-3-carboxylic acid (HMQC) was procured from avantor (USA) (https://www.avantorsciences.com), and its chemical structure is depicted in Scheme 1. The following metal salts were used for all selectivity and binding experiments: FeCl₃, CuCl₂, NiCl₂·6 H₂O, Zn(NO₃)₂·6 H₂O, Pb(NO₃)₂, CoCl₂·6 H₂O, MgSO₄, NaCl, CdCl₂, and Hg(NO₃)₂. All salts were of analytical grade and used without further purification, sourced from Sigma-Aldrich (https://www.sigmaaldrich.com). No further purification was performed on the chemicals prior to use. Acetic acid and sodium acetate were also purchased from Sigma-Aldrich, while 3-(N-morpholino)propanesulfonic acid (MOPS) and 3-(Cyclohexylamino)−2-hydroxy-1-propanesulfonic acid (CAPSO) were obtained from Sigma-Aldrich Co.

### Instrumentations

UV-Vis spectra were recorded using a Shimadzu UV-1800 UV/Visible Spectrophotometer (https://www.shimadzu.com), employing a quartz cell with a 1.0 cm path length. Luminescence spectra were obtained with a Jasco 6300 spectrofluorometer (https://jascoinc.com), also using a quartz cell with a 1.0 cm path length, and a 150 W xenon lamp for excitation. The excitation and emission bandwidths were set to 5 nm with medium sensitivity. The iron content in water samples was determined using a PerkinElmer Model 2380 Atomic Absorption Spectrophotometer, equipped with a hollow cathode lamp for iron. A digital pH meter (3510 Jenway, Bibby Scientific Ltd, UK) was used to measure the pH of the solutions, calibrated with standard buffers at pH 7.00, 4.00, and 10.00 at 25.0 ± 1.0 °C.

### Solutions Preparation

1 mM stock solutions of metal ions (Hg²⁺, Fe³⁺, Cu²⁺, Ni²⁺, Zn²⁺, Pb²⁺, Co²⁺, Mg²⁺, Na⁺, and Cd²⁺) were prepared by dissolving the respective salts in deionized water. The pH of the solutions was adjusted using appropriate buffer solutions, including acetic acid, MOPS, or CAPSO buffers, each at a concentration of 10 mM (sum of acid and base concentrations). The 1 mM HMQC stock solution was prepared by dissolving a specific amount of HMQC in ethanol.

For studying the selectivity of the probe toward the heavy metals under investigation, working solutions were freshly prepared by mixing fixed volumes of the metal ion stock solutions with a 1.0 mL aliquot of 1.0 × 10^− 3^ M HMQC stock solution in a 10 mL volumetric flask. The final volume was adjusted by adding the appropriate buffer solution. Once the solutions were prepared, they were ready for measurement.

The analysis was conducted by monitoring changes in the luminescence intensity upon the addition of Hg²⁺ ions at different pH values (4, 5, 6, 7, 8, 9, 10) using the suitable buffer solutions for each pH range. The interaction between the probe and Hg²⁺ ions was investigated using the Stern–Volmer equation for quenching analysis and the normal fluorescence intensity for enhancement analysis. These methods allowed for the detailed study of the fluorescence response of the probe in the presence of Hg²⁺ under different conditions.

### Computational Investigation

To further characterize the structures of the complex between the sensor and Hg which should help in understanding of the quenching mechanism, we performed DFT calculations utilizing Gaussian09 software. The geometries were successfully characterized using B3LYP functional in conjunction with 6–31 + G(d) basis sets for all atoms except Hg(II) metal ion which was treated using Lanl2DZ basis sets. In fact, the combination of B3LYP functional and 6–31 + G(d) basis sets has been used extensively to characterize the geometry of various chemical systems containing metals [31–33]. DFT calculations were performed on a simplified 1:1 HMQC–Hg²⁺ model in the gas phase, excluding explicit solvent and counteranions, in order to isolate and analyze the core electronic interactions involved in fluorescence modulation. Later, frequency analysis was carried out on the optimized chemical geometry to further underline the identity of the structures as stationary points with no imaginary frequency found.

### Quantum Yield Measurement

The relative fluorescence quantum yield (Φ) of HMQC was determined using a comparative method with quinine sulfate in 0.1 M H₂SO₄ (Φ = 0.546) as the reference standard. The measurements were performed in MOPS buffer (pH 7.0) at room temperature using a Jasco FP-6300 spectrofluorometer.

Both the standard and HMQC solutions were prepared with absorbance values below 0.1 at the excitation wavelength (λ_ex_ = 345 nm) to minimize inner filter effects. The quantum yield was calculated using the following Eq. (1):

1 $$\:{Q}_{x}={Q}_{st}\times\:\:\left(\frac{{\mathrm{I}}_{\mathrm{x}}}{{\mathrm{I}}_{\mathrm{s}\mathrm{t}}}\right)\times\:\:\left(\frac{{\mathrm{A}}_{\mathrm{s}\mathrm{t}}}{{\mathrm{A}}_{\mathrm{x}}}\right)\:\times\:\:\left(\frac{{\mathrm{n}}_{\mathrm{x}}^{2}}{{\mathrm{n}}_{\mathrm{x}}^{2}}\right)$$

Where:

- Φ_x_ is the quantum yield of HMQC,
- Φ_st_ is the quantum yield of the standard (quinine sulfate),
- *I* is the integrated fluorescence intensity,
- *A* is the absorbance at the excitation wavelength,
- *n* is the refractive index of the solvent.

### Fluorescence Determination Procedure of Hg(II)

The luminescence spectra and intensities were recorded at a fixed analytical emission wavelength of 415 nm for HMQC in both acetic acid buffer at pH 5.0 and MOPS buffer at pH 7. The excitation wavelength was set to 345 nm for all measurements. Fluorescence titrations were performed in a 1.0 cm quartz cuvette by gradually adding Hg²⁺ ions to a 100 µM HMQC solution. The fluorescence emissions were measured after incubating the solution for 5 min at room temperature. The measurements were repeated three times for each concentration, and the average fluorescence intensity was calculated to ensure accuracy. A calibration curve was constructed for both pH conditions. Unless otherwise noted, all metal ions were added at a final concentration of 50 nM, corresponding to 0.001 equivalents relative to the 50 µM HMQC solution used in the fluorescence measurements. For pH 5, the Stern–Volmer equation was used to analyze the data, relating the fluorescence intensity to the concentration of Hg²⁺. At pH 7, the relationship between fluorescence intensity and Hg²⁺ concentration was derived to build a calibration curve based on MOPS buffer. This procedure allowed for the precise quantification of mercury ions by monitoring the fluorescence response of the HMQC probe at both pH levels.

### Detection of Hg(II) in Different Water Samples

Four water samples were collected from Ismailia city, Egypt, and pretreated for analysis. The water samples were first filtered using a microfiltration membrane to remove suspended particles, and the pH was then adjusted to 7.4 using appropriate buffer solutions. A series of Hg²⁺ concentrations (10, 20, 30, 40, and 50 nM) were spiked into 1 mL of each water sample, along with 1 mL of 100.0 µM HMQC chemosensor solution. The fluorescence intensity was recorded after a 5-minute incubation at room temperature, using a 1.0 cm quartz cuvette. The procedure was repeated three times for each water sample to ensure the reliability of the measurements. The concentration of Hg²⁺ in the water samples was determined by analyzing the fluorescence data and referencing the calibration curve derived from the HMQC probe. This approach provided an efficient and accurate method for quantifying Hg²⁺ levels in real water samples, demonstrating the feasibility of using HMQC as a reliable chemosensor for environmental monitoring.

## Results and Discussion

### HMQC as a Chemosensor

The chemosensor HMQC (methyl 4-hydroxy-2-oxo-1,2-dihydroquinoline-3-carboxylic acid) is a versatile fluorescent molecule designed for selective detection of heavy metal ions in aqueous solutions [34]. Its chemical structure incorporates key functional groups, including a hydroxyl (-OH), a carboxyl (-COOH), a carbonyl group (-C = O), and an aromatic quinoline backbone [35]. These functional groups provide both binding sites for metal ions and a dynamic system for protonation-deprotonation reactions, enabling the molecule to exhibit significant optical changes under varying environmental conditions [36]. The aromatic backbone of HMQC allows for strong π-conjugation, facilitating electronic transitions that are highly responsive to its chemical environment [37]. The hydroxyl and carboxyl groups act as potential coordination sites for metal ions, where binding interactions can modulate the fluorescence emission. Heavy metal ions such as Hg²⁺ often induce fluorescence quenching due to processes like photoinduced electron transfer (PET) or heavy-atom effects [38]. In contrast, fluorescence enhancement may occur if the coordination stabilizes the molecule’s electronic excited state, reducing non-radiative decay pathways [39]. This dual behavior makes HMQC a promising candidate for sensing applications [40]. In addition to its metal-ion sensing capabilities, HMQC’s pH sensitivity expands its range of applications. The protonation-deprotonation equilibrium of its functional groups significantly alters the electron density and conjugation within the molecule, which in turn affects its photophysical properties [41]. Such sensitivity allows HMQC to act as a dual-functional chemosensor, capable of monitoring pH and detecting heavy metals in aqueous environments with high selectivity and sensitivity [42].

### UV-Vis Absorption Spectra Analysis of HMQC

**Fig. 1:**
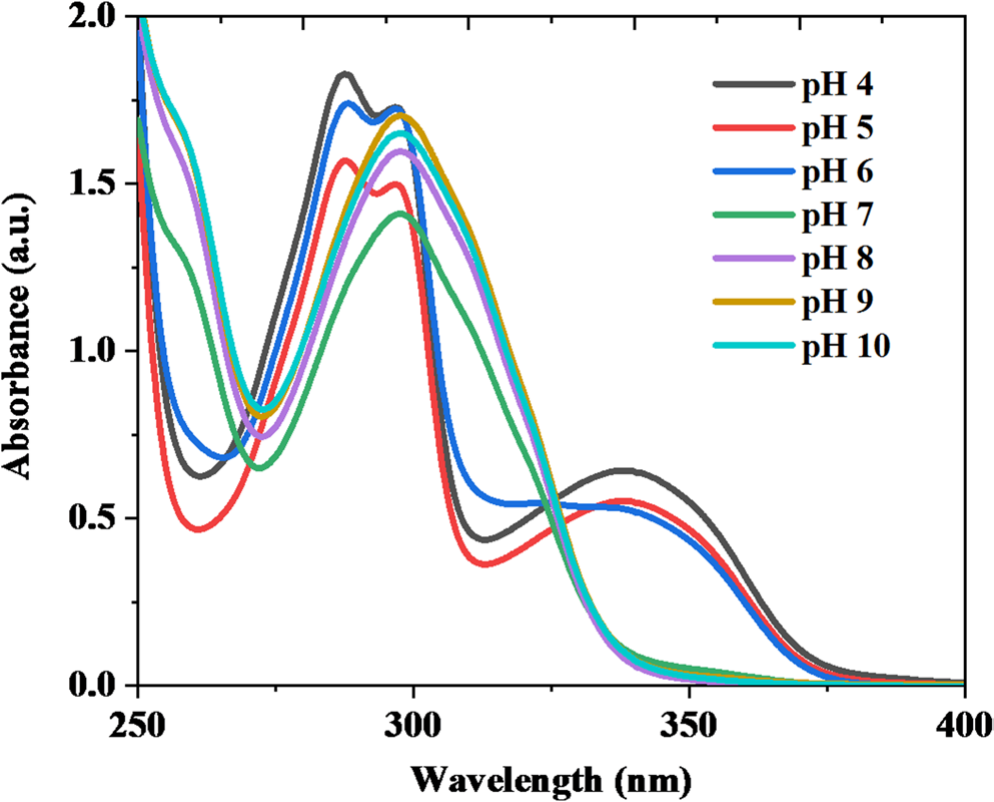
Uv-Vis absorption spectra of (5 × 10^− 5^ M) HMQC in different buffer solutions of pH range from 4–10 at room temperature

The UV-Vis absorption spectra of HMQC (5 × 10⁻⁵ M) were recorded in buffer solutions across a pH range of 4 to 10 at room temperature (Fig. 1), and significant spectral changes were observed. Under acidic to slightly neutral conditions (pH 4, 5, and 6), the spectra exhibited distinct absorption peaks at 287 nm, accompanied by a shoulder at 297 nm, and another peak at 339 nm. These peaks correspond to π-π* and n-π* electronic transitions. The π-π* transition originates from the aromatic and conjugated systems in HMQC, while the n-π* transition is attributed to lone-pair electrons on oxygen or nitrogen interacting with the conjugated π-system. As the pH increases from 7 to 10, the peaks at 287 nm and 339 nm vanish, leaving only the peak of the shoulder at 297 nm, which exhibits significant changes in intensity. Specifically: from pH 7 to 9, the absorbance at 297 nm increases, suggesting enhanced electron delocalization due to deprotonation of the hydroxyl (-OH) and carboxyl (-COOH) groups. At pH 10, a slight decrease in absorbance at 297 nm is observed, possibly due to a shift in equilibrium or reduced molecular stability in highly basic conditions. These spectral changes reflect structural modifications in HMQC caused by protonation-deprotonation processes. Under acidic conditions (pH 4–6), HMQC is predominantly in its protonated form, where both hydroxyl and carboxyl groups remain undissociated, stabilizing the π-conjugated system and supporting transitions at 287 nm and 339 nm. In contrast, at higher pH values (7–10), deprotonation disrupts the conjugation and modifies the electron density, leading to the disappearance of these peaks and the prominence of the absorption shoulder at 297 nm. The observed UV-Vis spectral behavior highlights the dual transitions in HMQC: π-π* transitions dominate in the protonated state due to the intact conjugated system. n-π* transitions contribute significantly, particularly in the neutral to slightly basic pH range, reflecting interactions involving lone pairs from oxygen and nitrogen atoms.

### Fluorescence Spectra Analysis of HMQC

**Fig. 2:**
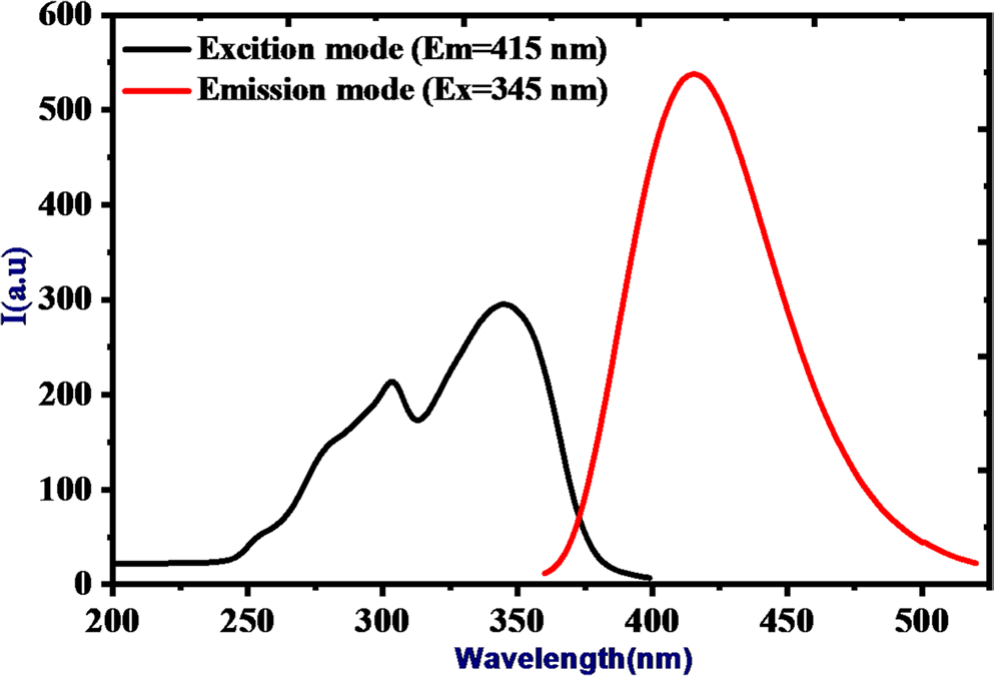
Excitation and emission fluorescence spectra of (5 × 10^− 5^ M) HMQC in MOPS buffer pH = 7 and at room temperature with medium sensitivity

Figure 2 illustrates the excitation and emission fluorescence spectra of HMQC (5 × 10⁻⁵ M) in a MOPS buffer solution at pH 7 and room temperature, recorded with medium sensitivity. The excitation spectrum, monitored at 415 nm, reveals a prominent peak centered at 345 nm, indicative of π-π* transitions within the molecule’s conjugated system. The emission spectrum, upon excitation at 345 nm, displays a broad fluorescence peak centered at 415 nm, reflecting radiative decay from the excited state to the ground state. A significant Stokes shift of approximately 70 nm is observed, highlighting efficient energy relaxation processes influenced by solvent interactions and structural stabilization in the excited state. The strong fluorescence emission at pH 6 underscores the stability of HMQC in its slightly acidic protonated form, with minimal non-radiative decay pathways. These properties, coupled with the well-separated excitation and emission wavelengths, make HMQC an effective fluorescent chemosensor, suitable for detecting environmental changes or interacting with analytes in aqueous media.

**Fig. 3:**
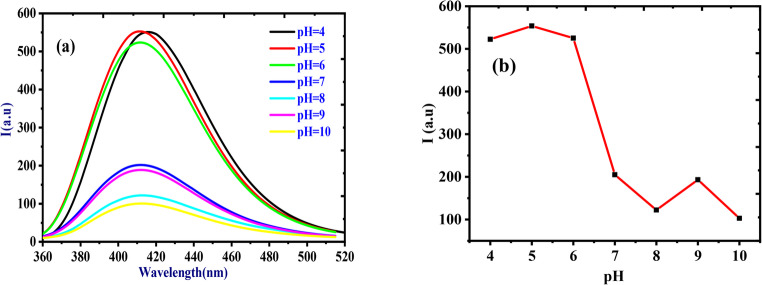
(**a**) Fluorescence spectra and (**b**) The effect of pH on the fluorescence intensity of (5 × 10− 5 M) HMQC in different buffer solutions of pH range from 4–10, at λex/em = 345/415 nm, at room temperature with medium sensitivity

Figure 3 illustrates the fluorescence spectra and pH dependence of HMQC (5 × 10⁻⁵ M) in buffer solutions across a pH range of 4–10, measured at an excitation wavelength of 345 nm and an emission wavelength centered at 415 nm under room temperature conditions. The fluorescence spectra (Fig. 3a) exhibit a prominent emission peak at 415 nm, with the intensity varying significantly based on the pH of the solution. At acidic pH values (4–6), the fluorescence intensity is at its highest, reaching a maximum at pH 5, where HMQC is predominantly in its protonated form. This protonation stabilizes the π-conjugated system, facilitating efficient radiative decay pathways. The intensity slightly decreases at pH 4 and 6 but remains relatively high. However, as the pH increases beyond 6, the fluorescence intensity sharply declines, as shown in Fig. 3b. This drop corresponds to the onset of deprotonation, particularly of the carboxyl (-COOH) group, which disrupts the conjugated electronic system and activates non-radiative decay pathways. At pH values of 7–10, the fluorescence intensity remains low, with minor fluctuations, reflecting the destabilization of the electronic structure in the deprotonated state. The bell-shaped fluorescence response highlights HMQC’s sensitivity to protonation states, making it a highly effective pH-sensitive fluorescent chemosensor. The sharp decrease in intensity between pH 6 and 7 underscores its utility in detecting subtle pH changes, particularly in slightly acidic to neutral environments, with potential applications in environmental monitoring, biological sensing, and analytical chemistry.

### Analysis of Fluorescence and Absorption of HMQC with Hg²⁺ at Different pH Levels

**Fig. 4:**
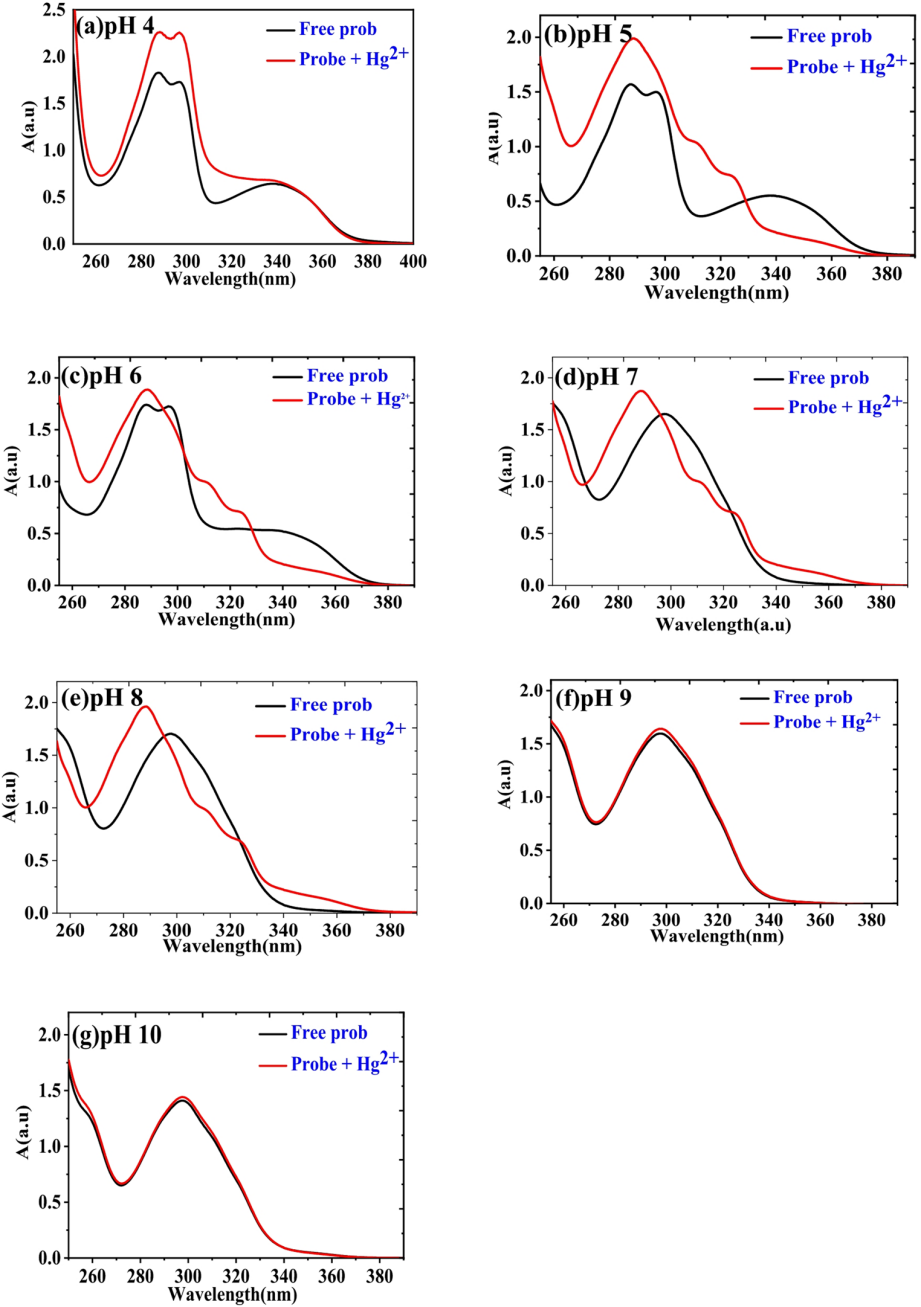
Absorption spectra of (5.0 × 10^− 5^ M; 1 equiv.) of HMQC with (5.0 × 10^− 5^ M; 1 equiv.) of Hg^2+^ in different pH range from 4–10 using buffer solutions

**Fig. 5:**
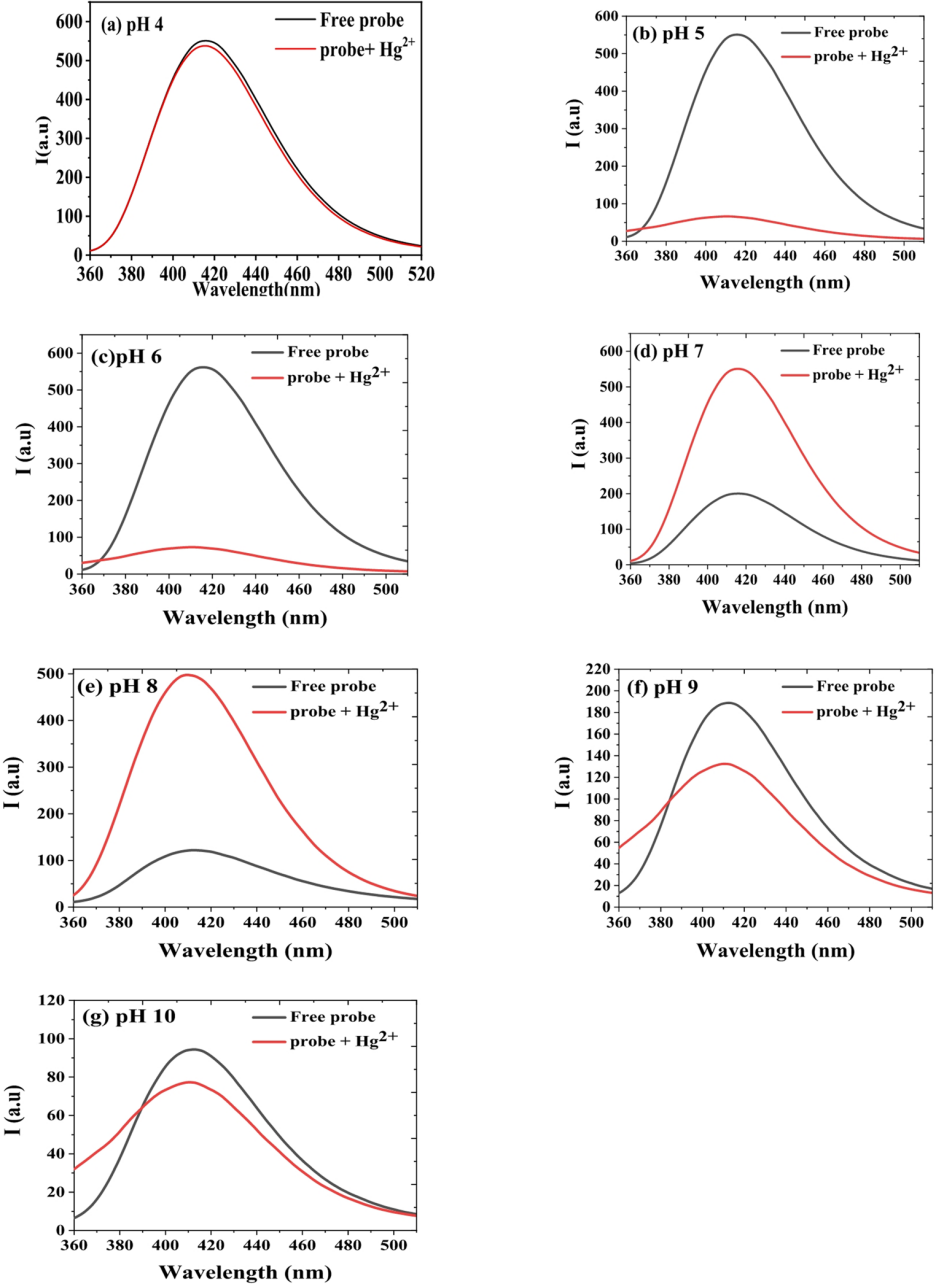
Fluorescence spectra of (5.0 × 10^− 5^ M; 1 equiv.) of HMQC with (5.0 × 10^− 5^ M; 1 equiv.) of Hg^2+^ in different pH range from 4–10 using buffer solutions at λ_ex_ = 345 nm, at room temperature

The interaction of HMQC with Hg²⁺ was studied through UV-Vis absorption and fluorescence spectra across a pH range of 4–10, revealing significant pH-dependent photophysical behavior. The results, as shown in Figs. 4 and 5, provide insights into the coordination mechanism between the probe and Hg²⁺, as well as the role of pH in modulating the probe’s response.

The UV-Vis absorption spectra (Fig. 4) demonstrate that the binding efficiency of HMQC to Hg²⁺ is strongest in acidic conditions (pH 4–6). At these pH values, the addition of Hg²⁺ results in an increase in absorbance at 287 nm, coupled with a red shift and broadening of the peak at 339 nm. These changes indicate effective coordination between the protonated functional groups of the probe (-OH and -COOH) and Hg²⁺, leading to perturbation of the electronic structure of HMQC. The largest absorption changes are observed at pH 5, highlighting this pH as the optimal condition for binding. Conversely, at pH 7 and above, the absorption spectra of the free and Hg²⁺-bound probe show minimal differences, with nearly identical spectra at pH 9 and 10. This behavior reflects reduced binding efficiency due to deprotonation of the functional groups, which limits the probe’s coordination ability.

The fluorescence spectra (Fig. 5) further confirm the pH-dependent behavior of the probe in the presence of Hg²⁺. At pH 5 and 6 (Fig. 5(a) and (b)), the addition of Hg²⁺ causes significant fluorescence quenching. This quenching is attributed to photoinduced electron transfer (PET) pathways, where Hg²⁺ binding facilitates non-radiative decay, reducing fluorescence intensity. The strongest quenching is observed at pH 5, aligning with the absorption data and reinforcing the conclusion that acidic pH values favor strong probe-Hg²⁺ interaction. At pH 7 and 8 (Fig. 5(c) and (d)), fluorescence enhancement is observed instead. This enhancement likely arises from a stabilization mechanism of the excited state, where Hg²⁺ binding suppresses non-radiative decay pathways and promotes radiative emission. The enhancement is most pronounced at pH 7, making it suitable for qualitative detection of Hg²⁺. However, at pH 9 and 10 (Fig. 5(e) and (f)), no significant fluorescence changes are observed upon Hg²⁺ addition, consistent with the absorption data that show negligible binding in basic conditions.

The combined analysis of Figs. 4 and 5 underscores the dual fluorescence response of HMQC to Hg²⁺, which is governed by pH. At acidic pH (4–6), the quenching effect provides a robust mechanism for quantitative detection, with a well-defined linear relationship between fluorescence intensity and Hg²⁺ concentration. Meanwhile, at neutral pH (7–8), fluorescence enhancement offers a visually distinct signal, making it ideal for qualitative screening in neutral environments such as natural or drinking water. The pH-dependent behavior highlights the probe’s adaptability for use in diverse analytical scenarios, with optimal performance in acidic environments for precise quantification and in neutral environments for rapid detection. This versatility makes HMQC a promising tool for Hg²⁺ monitoring in environmental and water quality applications.

To further validate the fluorescence enhancement mechanism of HMQC upon Hg²⁺ binding under different pH conditions, we evaluated the fluorescence quantum yield at pH 7.0, where the probe exhibited optimal emission. The relative quantum yield was calculated using quinine sulfate in 0.1 M H₂SO₄ as a reference standard (Φ = 0.546), following established fluorescence protocols. In the absence of Hg²⁺, HMQC showed a quantum yield of 0.21, which increased significantly to 0.43 upon the addition of 50 nM Hg²⁺. This notable enhancement in radiative efficiency supports the proposed “turn-on” fluorescence mechanism, confirming the formation of a stable HMQC–Hg²⁺ complex. These findings are in good agreement with the observed emission spectra and further reinforce the suitability of HMQC as a selective and sensitive chemosensor for mercury(II) detection in mildly acidic to neutral environments.

HMQC’s structural versatility, combined with functional groups capable of coordinating heavy metal ions, enables selective fluorescence responses in the presence of Hg²⁺. The metal-ion sensing capabilities makes HMQC a highly valuable tool for applications in environmental monitoring, biological systems, and analytical chemistry.

### Binding Stoichiometry

**Fig. 6:**
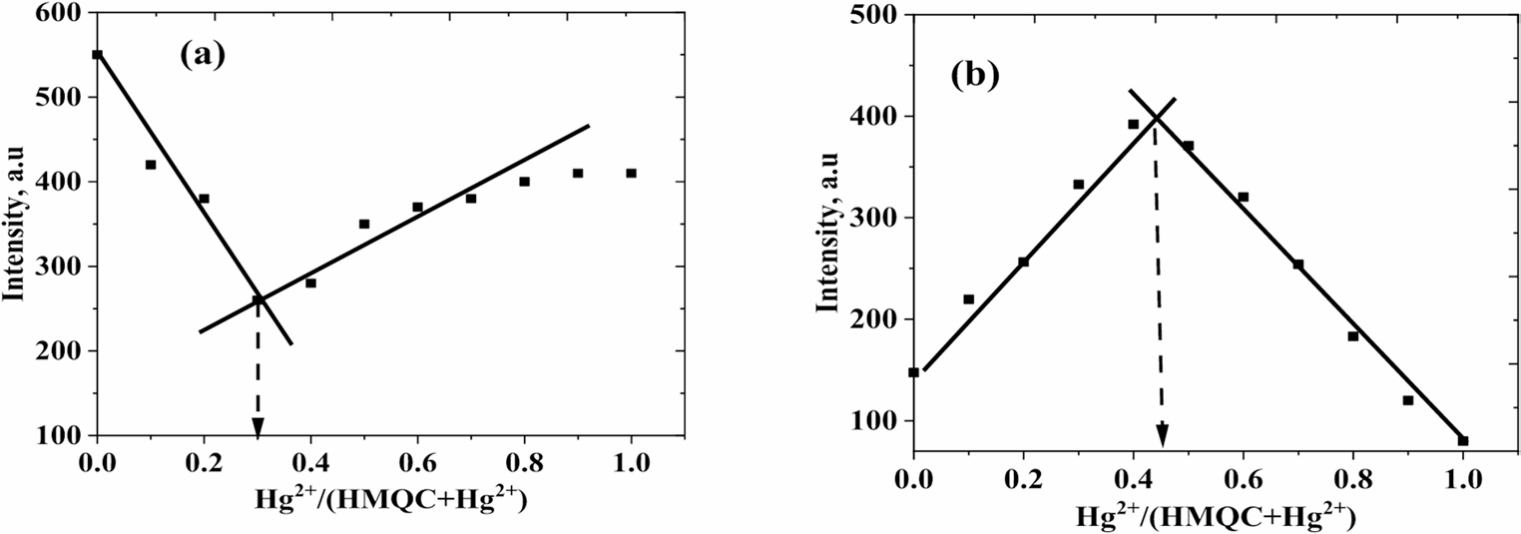
Job’s plot of a HMQC for varying mole fraction of Hg^2+^in (**a**) acetic acid buffer (pH 5.0) and (**b**) MOPS buffer (pH = 7.0), λ_ex/em_ = 345/415 nm at room temperature

The binding stoichiometry between HMQC and Hg²⁺ was determined using Job’s plot analysis, as shown in Fig. 6. The experiments were conducted at two different pH values: pH 5.0 in acetic acid buffer (Fig. 6(a)) and pH 7.0 in MOPS buffer (Fig. 6(b)). The fluorescence intensity at 415 nm was recorded as a function of the mole fraction of Hg²⁺ at a fixed total concentration of the probe and Hg²⁺. It is important to note that Job’s plot analysis is designed solely to determine the binding stoichiometry under fixed total concentration conditions and does not reflect fluorescence saturation behavior. Therefore, the absolute emission intensities obtained from Job’s plot experiments should not be directly compared with those obtained from fluorescence titration or calibration experiments.

At pH 5.0, the Job’s plot reveals an intersection at a mole fraction of Hg²⁺ of approximately 0.33, indicating a 2:1 binding stoichiometry between HMQC and Hg²⁺. This stoichiometry suggests that two molecules of HMQC coordinate with one Hg²⁺ ion. The quenching of fluorescence observed in this pH condition is consistent with the strong interaction between Hg²⁺ and the protonated hydroxyl (-OH) and carboxyl (-COOH) groups of the probe, which provide multiple coordination sites. The higher protonation state of the functional groups at acidic pH facilitates this multidentate binding, leading to the formation of a stable 2:1 complex. In contrast, at pH 7.0, the Job’s plot shows an intersection at a mole fraction of Hg²⁺ of approximately 0.50, indicating a 1:1 binding stoichiometry between HMQC and Hg²⁺. Under these neutral conditions, the partial deprotonation of the functional groups reduces the number of available binding sites, resulting in a 1:1 complex. The fluorescence enhancement observed at pH 7.0 correlates with this stoichiometry, where the binding of one Hg²⁺ ion to a single HMQC molecule stabilizes the probe’s excited state and reduces non-radiative decay pathways. These findings demonstrate the pH-dependent nature of the interaction between HMQC and Hg²⁺. At acidic pH, the higher degree of protonation facilitates the formation of a 2:1 complex, whereas at neutral pH, the reduced protonation state limits the coordination to a 1:1 complex. This pH-dependent stoichiometry aligns with the observed fluorescence behavior—quenching at pH 5.0 and enhancement at pH 7.0—and highlights the versatility of HMQC as a chemosensor for Hg²⁺ detection in diverse environmental conditions.

### Reversibility

**Fig. 7:**
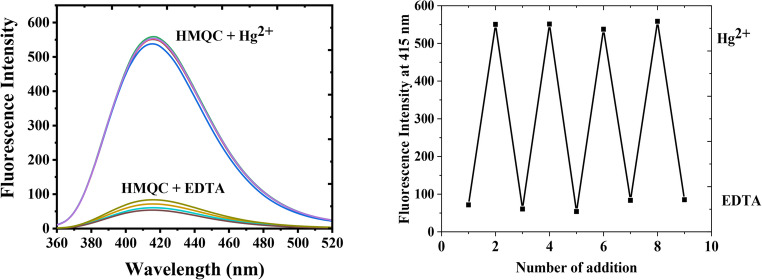
Fluorescence reversibility study of HMQC (5.0 × 10⁻⁵ M; 1.0 equiv.) in MOPS buffer (pH 7.0) at room temperature. Addition of Hg²⁺ (50 nM; 0.001 equiv.) at 415 nm (λₑₓ = 345 nm), followed addition of EDTA (50 nM; 0.001 equiv.)

The reversibility of HMQC’s fluorescence response to Hg²⁺ was evaluated by alternately adding Hg²⁺ (50 nM) and EDTA to the solution. Figure 7 illustrates the results of this study, showcasing the fluorescence spectra (Fig. 7(a)) and the fluorescence intensity at 415 nm as a function of the number of additions (Fig. 7(b)). The addition of Hg²⁺ to the HMQC solution resulted in a significant increase in fluorescence intensity at 415 nm, consistent with the enhancement observed in the presence of Hg²⁺ due to excited-state stabilization. Subsequent addition of EDTA caused a sharp decrease in fluorescence intensity, reflecting the displacement of Hg²⁺ from the HMQC-Hg²⁺ complex by EDTA. This occurs because EDTA forms a more stable chelation complex with Hg²⁺, effectively removing it from the probe. The repeated cycling between Hg²⁺ and EDTA additions demonstrates a reversible fluorescence response, with the system returning to its initial intensity levels upon the introduction of EDTA. The fluorescence intensity at 415 nm was monitored over eight sequential additions of Hg²⁺ and EDTA. The cyclic trend in fluorescence intensity shows consistent enhancement upon Hg²⁺ addition and quenching upon EDTA addition. The reproducibility of this cyclic response indicates that the interaction between HMQC and Hg²⁺ is fully reversible. The probe retains its functionality over multiple binding and displacement cycles, highlighting its robustness and suitability for real-time sensing applications. The reversibility study confirms that HMQC interacts with Hg²⁺ through a dynamic and non-permanent binding mechanism, allowing it to respond reversibly to changes in the presence of Hg²⁺. The ability of EDTA to displace Hg²⁺ from the HMQC-Hg²⁺ complex highlights the competitive binding behavior of the system, where Hg²⁺ preferentially forms a complex with EDTA. This reversibility is a critical feature for practical applications, as it allows the probe to be reused for continuous or repeated sensing of Hg²⁺. The results in Fig. 7 demonstrate that HMQC exhibits excellent reversibility in its fluorescence response to Hg²⁺. This property enhances the probe’s potential for real-time monitoring and repetitive sensing of Hg²⁺ in environmental and analytical applications. The dynamic nature of the interaction between HMQC and Hg²⁺, coupled with its reversible fluorescence response, underscores its utility as a robust and reusable chemosensor for mercury detection.

### Effect of Response time

**Fig. 8:**
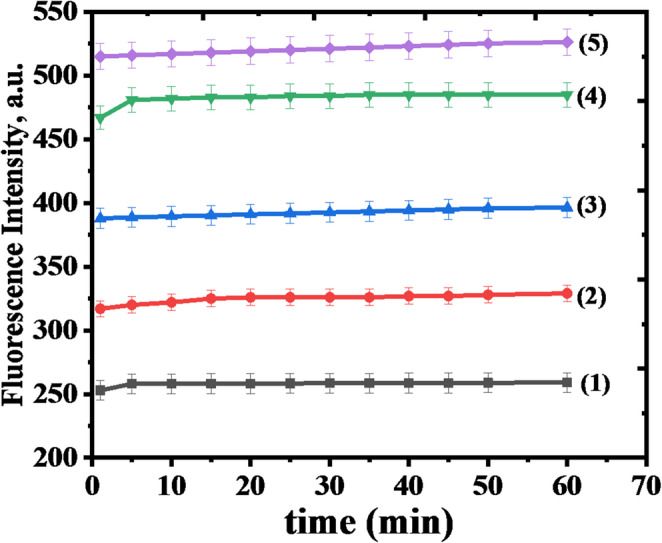
Fluorescence response of (5.0 × 10^− 5^ M) HMQC in presence of a Hg^2+^ with different concentrations at different times in MOPS buffer pH = 7. The concentrations of Hg^2^ are: (1) 10.0, (2) 20.0, (3) 30.0, (4) 40.0, (5) 50.0 (nM) respectively

The fluorescence response of HMQC to Hg²⁺ was evaluated over time by monitoring the fluorescence intensity at 415 nm for various Hg²⁺ concentrations ranging from 10 nM to 50 nM, as shown in Fig. 8. For all tested concentrations, the fluorescence intensity stabilized within the first 5 min of measurement, indicating fast binding kinetics and the rapid establishment of equilibrium between HMQC and Hg²⁺. This rapid stabilization highlights 5 min as the optimal measurement time for achieving reliable and accurate fluorescence readings. The fluorescence intensity remained stable over a 60-minute period, with no noticeable photobleaching or degradation, underscoring the robustness and reliability of the HMQC-Hg²⁺ complex for continuous monitoring. The concentration-dependent fluorescence enhancement further demonstrates the probe’s high sensitivity and dynamic response range for Hg²⁺ detection. These results establish HMQC as a fast, stable, and efficient chemosensor, capable of providing sensitive and reliable detection of Hg²⁺ in real-time applications, with 5 min being the recommended measurement time.

### Effect of Hg(II) Concentrations

**Fig. 9:**
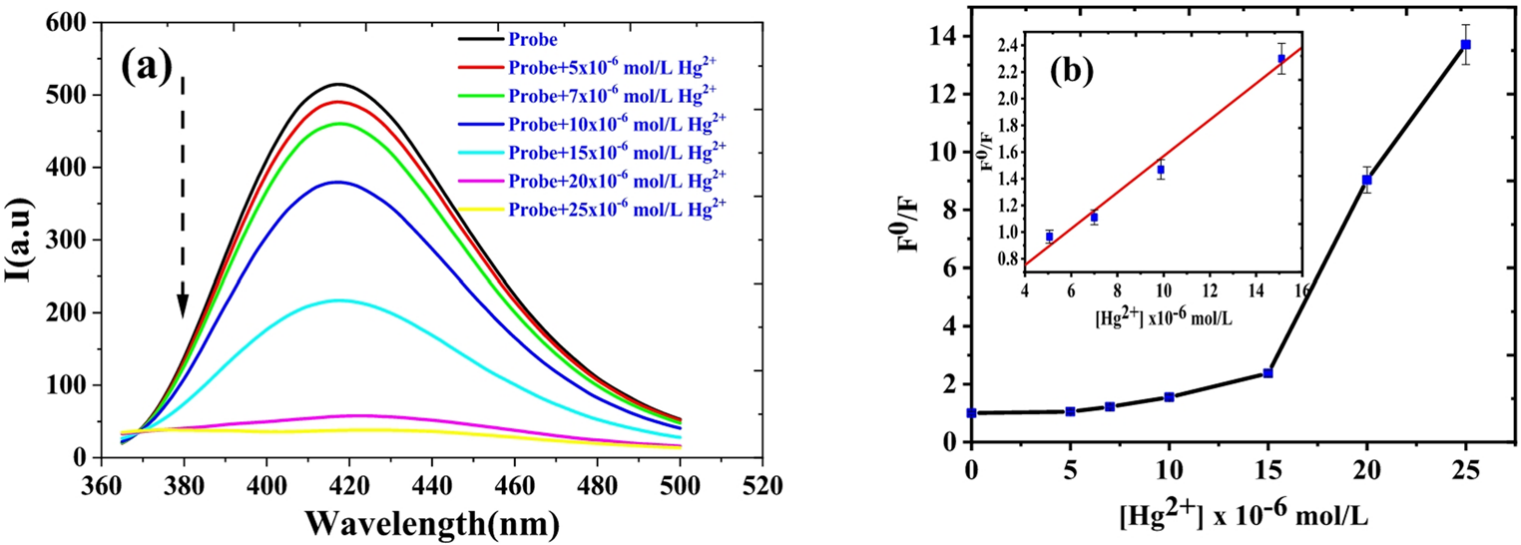
(**a**) Fluorescence spectra and (**b**) Stern Volmer plot for (5.0 × 10^− 5^ M) HMQC with different concentration of Hg^2+^ in acetic acid buffer (pH = 5), λ_ex_ = 345 nm and at room temperature

**Fig. 10:**
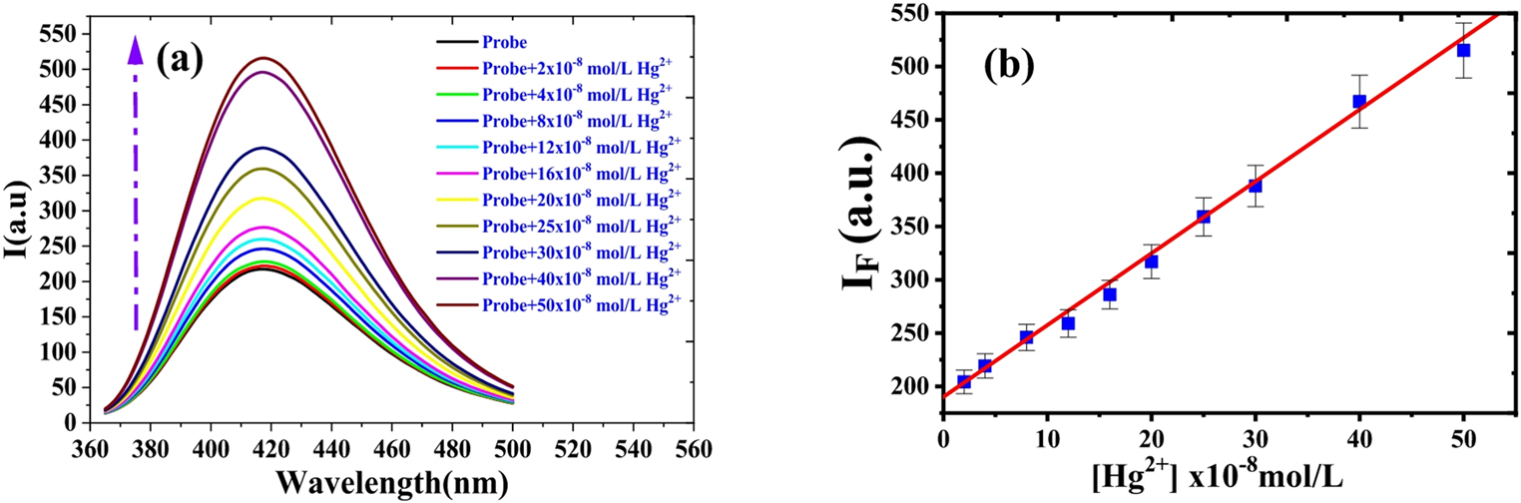
(**a**) Fluorescence spectra and (**b**) calibration curve for (5.0 × 10^− 5^ M) HMQC with different concentration of Hg^2+^ in MOPS buffer (pH = 7), λ_ex_ = 345 nm and at room temperature

The calibration data for the HMQC probe with Hg²⁺ were analyzed under two pH conditions: acetic acid buffer (pH 5) and MOPS buffer (pH 7). The results, illustrated in Figs. 9 and 10, and summarized in Table 1, demonstrate the probe’s fluorescence response, sensitivity, and linearity under these conditions, providing insights into its optimal performance for mercury detection.

At pH 5 (Fig. 9), the fluorescence spectra show a clear quenching effect with increasing Hg²⁺ concentrations, consistent with the Stern–Volmer quenching mechanism. The corresponding Stern–Volmer plot in Fig. 9(b) follows the equation F0/F = 0.73 + 0.13[Hg^2+^] (µM), with a linear range of 3.09–15 µM. The limit of detection (LOD) was determined to be 0.93 µM, and the limit of quantification (LOQ) was 3.09 µM. While this calibration is suitable for environments with high Hg²⁺ concentrations, such as acidic industrial effluents, the relatively high LOD and LOQ make it less effective for trace-level detection. Furthermore, the sharp decrease in fluorescence intensity observed upon the addition of 15 µM Hg²⁺ (Fig. 9) can be rationalized by considering the coordination dynamics at acidic pH. Initially, the HMQC chemosensor forms a stable 1:2 complex with Hg²⁺, leading to effective fluorescence quenching via photoinduced electron transfer (PET) and other non-radiative decay pathways. However, as the concentration of Hg²⁺ increases beyond a 2:1 Hg²⁺:HMQC molar ratio, excess Hg²⁺ ions may begin to interact nonspecifically or induce aggregation or secondary complex formation. This results in structural distortions or over-coordination, disrupting the π-conjugated system and increasing non-radiative relaxation. The inflection point seen in the Stern–Volmer plot further supports this hypothesis, marking a transition from dynamic to static quenching. Similar quenching phenomena at high metal ion concentrations have been reported in other fluorescence-based sensing systems [17, 43, 44].

At pH 7 (Fig. 10), the addition of Hg²⁺ induces a strong turn-on fluorescence response as the deprotonated form of HMQC (–O⁻/–COO⁻) coordinates to the metal ion, forming the rigid 1:1 chelate responsible for suppressing the ICT quenching pathway and activating the CHEF mechanism. In the low-concentration regime, the emission intensity increases proportionally to the Hg²⁺ concentration, and the calibration curve follows the relationship: F = 189.13 + 6.58 [Hg²⁺] (nM) with a linear dynamic range of 4.84–50 nM, an LOD of 1.47 nM, and an LOQ of 4.84 nM, demonstrating excellent analytical sensitivity. A key observation is that the fluorescence intensity reaches a plateau at concentrations higher than ~ 50 nM, even though Hg²⁺ was further increased up to the micromolar range. This phenomenon is not inconsistent with the 1:1 binding stoichiometry but instead reflects the photophysical saturation of the emissive pathway. According to fluorescence-binding theory (Valeur, Lakowicz), when the apparent binding constant (Ka) is high—as is typical for soft metal ions such as Hg²⁺ interacting with deprotonated oxygen donors—the emissive complex HMQC–Hg²⁺ is formed efficiently at very low metal-ion concentrations. Because CHEF amplification does not scale linearly with the total amount of complex but rather with the fraction of emissive chromophores formed, full optical response can be achieved even when only a small proportion of the total probe molecules participate in the high-affinity complex. Beyond this point, additional Hg²⁺ may still interact with excess ligand, but these interactions occur either: at non-emissive secondary sites, in geometries that do not suppress ICT, or through weak outer-sphere interactions dominated by solvent/counterion competition. These pathways do not contribute to fluorescence enhancement and therefore do not increase the signal, causing the observed plateau. This behavior is well documented in high-affinity chelating fluorophores, where the fluorescence output is governed by the saturation of the emissive binding site rather than by overall stoichiometric equivalence [45–48]. It should be emphasized that this fluorescence plateau reflects photophysical (optical) saturation of the emissive CHEF-active Hg²⁺–HMQC complex rather than complete stoichiometric saturation of all available ligand molecules in solution. The combination of a low linear range threshold, a strong CHEF response, and early optical saturation collectively confirms that the 1:1 Hg–HMQC complex formed at neutral pH is both photophysically dominant and energetically preferred. This behavior further validates the suitability of the sensor for ultra-trace mercury detection in environmental applications. Figure 10 illustrates the analytical calibration of Hg²⁺ at pH 7 within the low-concentration regime (4.84–50 nM), which is the most relevant range for trace-level mercury detection and for determining key analytical parameters such as the linear range, limit of detection (LOD), and limit of quantification (LOQ). In this concentration window, the fluorescence intensity increases linearly with increasing Hg²⁺ concentration. Beyond approximately 50 nM, the fluorescence response reaches a plateau, which does not indicate that bulk stoichiometric equivalence between Hg²⁺ and HMQC has been achieved. Rather, this behavior reflects photophysical saturation of the emissive pathway arising from the very high binding affinity of Hg²⁺ toward the deprotonated HMQC ligand at neutral pH (Kₐ = 1.12 × 10⁶ L·mol⁻¹). Under such high-affinity conditions, conversion of the fraction of probe molecules responsible for fluorescence enhancement into the emissive chelated form occurs efficiently at low Hg²⁺ concentrations, after which further increases in Hg²⁺ concentration do not result in additional emission enhancement. The use of Hg²⁺ concentrations up to 5.0 × 10⁻⁵ M, as shown in Fig. 5d, was intended to confirm that the system had fully entered the signal saturation regime by employing equimolar concentrations of Hg²⁺ and HMQC (50 µM each). While Fig. 10 establishes the quantitative analytical performance of the probe at ultra-trace Hg²⁺ levels, Fig. 5d demonstrates the robustness of the fluorescence plateau and confirms that no further enhancement occurs even under equimolar conditions. This distinction clarifies the complementary roles of the two figures and avoids confusion between analytical calibration and signal saturation behavior.

The data summarized in Table 1 clearly indicate that the probe performs better at pH 7 compared to pH 5. The improved sensitivity at pH 7 is reflected in the lower LOD and LOQ values, as well as the broader linear range, enabling accurate quantification of Hg²⁺ concentrations across a wider spectrum. This performance aligns closely with international regulatory standards for mercury in drinking water. According to the World Health Organization (WHO) and the European Union (EU) guidelines, the maximum allowable concentration of Hg²⁺ in drinking water is 1 µg/L (5 nM). The LOD of the HMQC probe at pH 7 (1.47 nM) is well below this limit, making it suitable for compliance monitoring and ensuring water safety.

Figures 9 and 10 further emphasize the pH-dependent behavior of HMQC, where quenching at pH 5 is ideal for high-concentration environments, while enhancement at pH 7 is more effective for trace-level detection. The broader linear range and higher sensitivity observed at pH 7 make it the preferred condition for subsequent analyses. All further studies and real-world applications of the HMQC probe were conducted at pH 7 to capitalize on these advantages and to align with water quality standards for mercury detection in environmental and drinking water samples.

**Table 1:** Calibration data of HMQC probe with Hg^2+^ in case acetic acid buffer solution (pH 5) and in case MOPS buffer solution (pH7)

Parameters	In case acetic acid buffer solution (pH 5)	In Case MOPS buffer solution (pH 7)
Regression equation	F^0^/F = 0.73 + 0.13 [Hg^2+^] (µM)	F = 189.13 + 6.58 [Hg^2+^] (nM)
Linear range	3.09–15.09 (µM)	4.84 −50 (nM)
R^2^	0.9954	0.9996
LOD	0.93 (µM)	1.47 (nM)
LOQ	3.09 (µM)	4.84 (nM)
Number of points	4	10

### Significance and Novelty of HMQC

The comparison in Table 2 highlights the exceptional performance and unique features of HMQC as a chemosensor for detecting mercury ions (Hg²⁺) in aqueous environments. Its superior sensitivity, dual detection mechanism, and practical adaptability underscore its potential for environmental monitoring and real-world applications.

HMQC demonstrates remarkable sensitivity, achieving a limit of detection (LOD) of 1.47 nM, which is significantly lower than most other sensors in the comparison, such as the Coumarin-Based Probe (20 nM) and Pyrido-Benzimidazole sensor (239 nM). This sensitivity meets and exceeds international regulatory standards, such as the World Health Organization (WHO) limit of 1 µg/L (5 nM) for mercury in drinking water. Such trace-level detection capability positions HMQC as a highly effective tool for monitoring mercury contamination in critical applications.

Unlike many other chemosensors that rely on a single detection mechanism, HMQC offers a dual fluorescence response, exhibiting quenching under acidic conditions (pH 5) and enhancement at neutral pH (pH 7). This versatility provides an adaptable platform for detecting Hg²⁺ in diverse environmental conditions, optimizing its performance across a range of real-world scenarios. Sensors with single-mechanism responses, such as Rhodamine-Derived sensors or Coumarin-Based Probes, lack this adaptability, which limits their utility in complex environments.

The operating medium further highlights the practicality of HMQC. It performs efficiently in MOPS and acetate buffers, which are commonly used in environmentally relevant studies. In contrast, several other sensors require organic solvents, such as THF or DMF, which are less suitable for environmental applications due to their toxicity and incompatibility with aqueous systems. This adaptability makes HMQC particularly valuable for applications involving natural water sources and drinking water samples.

Another critical advantage of HMQC is its demonstrated efficacy in real-world applications. The sensor was validated in real water samples, achieving recovery rates exceeding 92% and maintaining reproducibility with relative standard deviations (RSDs) below 5%. This practical validation distinguishes HMQC from other sensors, such as Coumarin-Rhodamine, which are limited to laboratory settings or specialized uses like bioimaging.

The dual fluorescence response of HMQC represents a novel contribution to the field of chemosensor design. Its ability to function as both a quencher and enhancer, depending on the pH conditions, offers a significant advancement in versatility and reliability. By addressing the limitations of existing sensors, HMQC provides a robust and flexible solution for mercury detection in environmental and public health contexts.

In summary, HMQC demonstrates significant potential as an advanced mercury chemosensor, combining high sensitivity, selectivity, and versatility for real-world applications. Its innovative dual fluorescence response and validated performance in environmental samples position it as a promising tool for mercury detection in both research and practical contexts.

**Table 2:** Comparison of recent chemosensors for Hg²⁺ detection

Sensor	Detection Mechanism	LOD	Medium (pH)	Applications	Reference
HMQC	Dual (Enhancement/Quenching)	1.47 nM	MOPS/Acetate	Real Water Samples	Current Work
Coumarin-Based Probe	Fluorescence quenching	20 nM	Acetate Buffer	Industrial Effluents	[23]
Rhodamine-Derived Sensor	Fluorescence enhancement	5 nM	MOPS Buffer (pH 7)	River Water	[10]
Rhodamine-based	Colorimetric	32 nM	CH₃CN: H₂O (1:1, v/v)	Logic Gates	[49]
Coumarin-Rhodamine	FRET-based	208 nM	H₂O/CH₃CN (1:4, v/v)	Bioimaging, Water Samples	[50]
Pyrido-Benzimidazole	Ratiometric Fluorescence	239 nM	H₂O (pH 7.0)	Environmental Detection	[51]
Diarylethene	Fluorometric	0.42 µM	THF: H₂O (7:3, v/v, pH 7.4)	Cell Imaging	[52]
DMF-Based Sensor	Fluorometric	195 nM	DMF: H₂O (1:1, v/v)	Fluorescence Indicator	[53]
Fluorescent Probe X	Turn-on Fluorescence	70 nM	DMSO: H₂O (9:1, pH 7.2)	Real-Time Detection	[54]

### Application of the Dual Fluorescence Response of the Probe in Hg²⁺ Detection

The probe’s dual fluorescence behavior—quenching at acidic pH (pH 5) and enhancement at neutral pH (pH 7)—presents a versatile platform for both qualitative and quantitative detection of Hg²⁺ in various environmental matrices. This pH-dependent behavior allows the probe to be applied effectively under different scenarios tailored to specific sample types and analytical needs.

At pH 5, the probe exhibits fluorescence quenching in the presence of Hg²⁺, attributed to photoinduced electron transfer (PET) mechanisms facilitated by the interaction of Hg²⁺ with the protonated functional groups of the probe. This quenching behavior enables precise quantification of Hg²⁺ through a well-defined linear relationship between the fluorescence intensity and Hg²⁺ concentration, making it ideal for quantitative analysis in acidic samples such as industrial wastewater. Conversely, at pH 7, the probe demonstrates fluorescence enhancement upon binding Hg²⁺, likely due to the stabilization of the excited state and suppression of non-radiative decay pathways. This enhancement provides a visually distinct and easily interpretable signal, suitable for qualitative detection and rapid screening in neutral environments such as drinking water or natural water sources.

To leverage this dual functionality, the probe can be utilized in a two-step analytical strategy. Initial qualitative detection can be performed at pH 7, where fluorescence enhancement allows rapid identification of Hg²⁺. Subsequently, quantitative analysis can be conducted at pH 5 to accurately determine the concentration of Hg²⁺ in the sample. This approach ensures both efficiency and accuracy, demonstrating the probe’s adaptability to diverse analytical scenarios.

In practical applications, the enhancement mode at pH 7 may be prioritized for environmental monitoring in neutral pH samples due to its straightforward signal response and ease of interpretation. Alternatively, the quenching mode at pH 5 is better suited for samples with low pH, such as acidic industrial effluents, where precise quantification is critical. By tailoring the detection mode to the specific sample matrix, the probe demonstrates its robustness and versatility as a reliable chemosensor for Hg²⁺ detection in environmental and water quality assessments.

### Selectivity

**Fig. 11:**
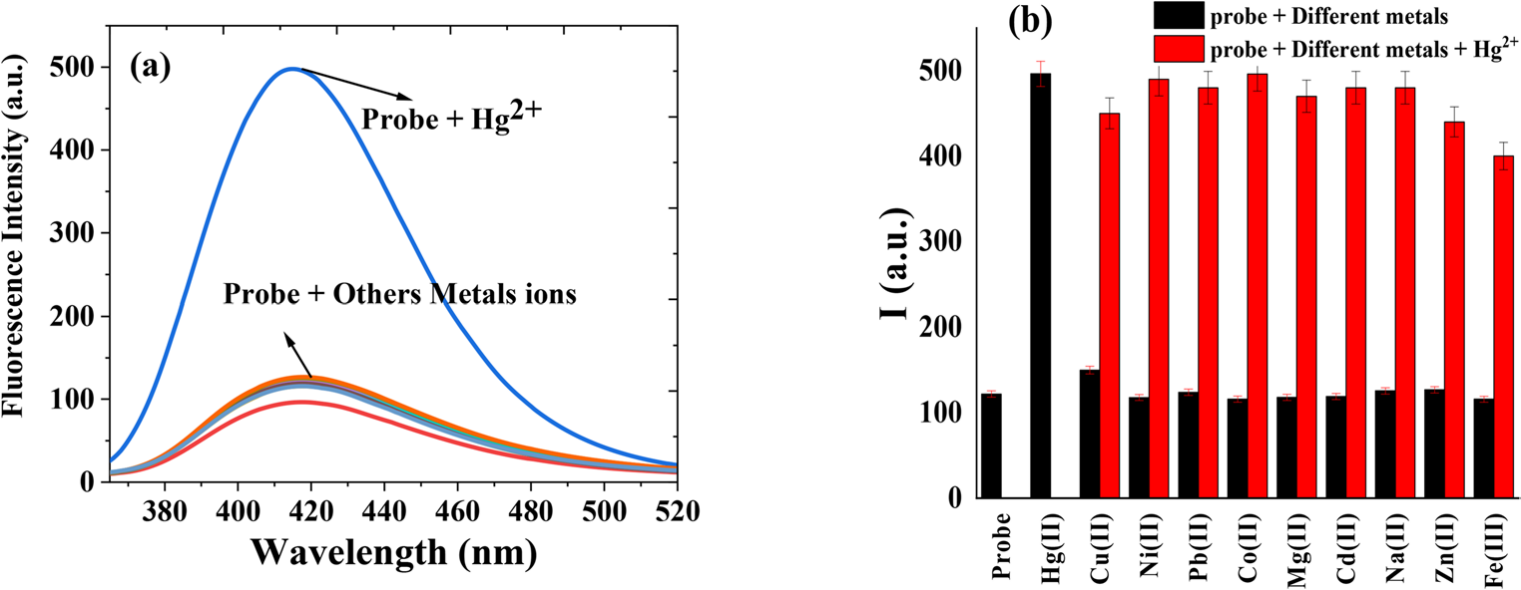
(**a**) Fluorescence spectra of (5.0 × 10^− 5^ M; 1.0 equiv.) HMQC with metal ions in MOPS buffer solution (pH = 7); (**b**) Selectivity of HMQC toward Hg^2+^ and (50 nM; 0.001 equiv.) other metal ions in absence (black bars) and presence of (50 nM; 0.001 equiv.) Hg^2+^ (red bars) at λ_ex/em_ = 345/415 nm in MOPS buffer solution pH 7 at room temperature

The selectivity experiments were conducted at neutral pH 7.0, as this condition corresponds to the turn-on fluorescence response of HMQC upon Hg²⁺ binding, which enables more reliable differentiation between target and interfering ions. At pH 5, although complexation with Hg²⁺ still occurs, the sensing mechanism shifts toward fluorescence quenching, making comparative analysis with other ions less conclusive. The selectivity of HMQC for Hg²⁺ over other metal ions were evaluated using fluorescence spectroscopy, as shown in Fig. 11. The experiments were conducted in MOPS buffer solution at pH 7 with a fixed probe concentration of 5.0 × 10^− 5^ M (1.0 equiv.), at λ_ex/em_ = 345/415 nm and at room temperature. In Fig. 11(a), the fluorescence spectra of HMQC in the presence of 50 nM (0.001 equiv.) of various metal ions (Cu²⁺, Pb²⁺, Ni²⁺, Cd²⁺, Co²⁺, Zn²⁺, and Fe³⁺) are compared with the spectrum obtained in the presence of Hg²⁺. The fluorescence intensity of HMQC shows negligible enhancement or change upon the addition of other metal ions, indicating minimal interaction between HMQC and these ions. However, upon the addition of Hg²⁺, a significant fluorescence enhancement is observed, demonstrating the high selectivity of HMQC for Hg²⁺ in a neutral pH environment. This distinct response to Hg²⁺ is attributed to the strong coordination between Hg²⁺ and the probe’s functional groups, leading to stabilization of the excited state and enhancement of fluorescence intensity. Figure 11(b) further illustrates the selectivity of HMQC by comparing fluorescence intensities in the presence of different metal ions (black bars) and a mixture of these metal ions with Hg²⁺ (red bars). The fluorescence intensity remains low for all metal ions when added individually, confirming their minimal interference with HMQC. However, when Hg²⁺ is introduced into the system, the fluorescence intensity increases significantly, even in the presence of competing metal ions. This behavior highlights the probe’s ability to selectively detect Hg²⁺ without interference from other metal ions, making it a highly selective chemosensor for Hg²⁺ detection.

These results collectively demonstrate the excellent selectivity of HMQC for Hg²⁺ under neutral pH conditions. The lack of significant interference from other common metal ions, even at equal concentrations, underscores the probe’s potential for practical applications in complex environmental matrices. The combination of strong fluorescence enhancement in the presence of Hg²⁺ and minimal response to other ions establishes HMQC as a reliable and selective chemosensor for mercury ion detection.

### DFT Results

**Fig. 12:**
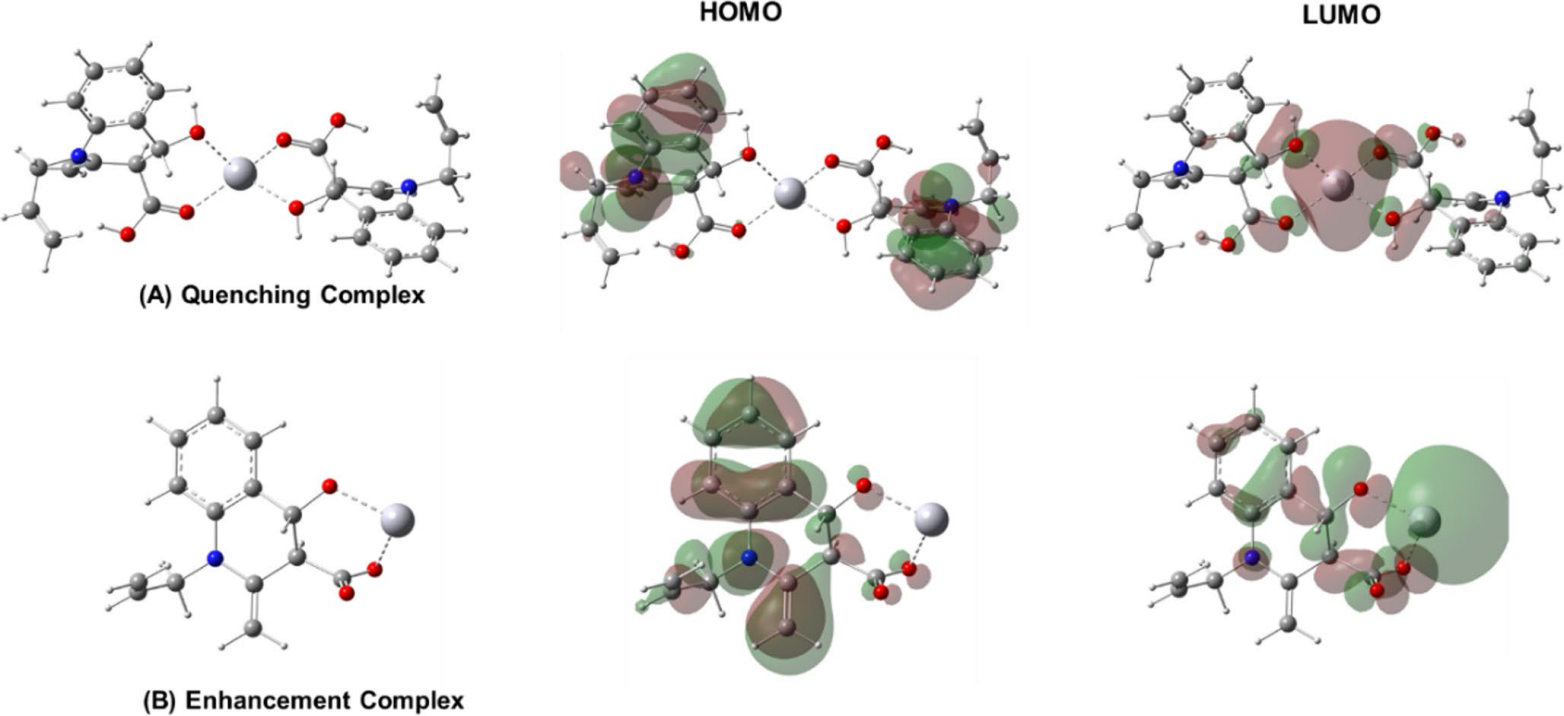
The optimized molecular geometries for the (**A**) Hg-HMQC fluorescence quenching (**B**) Hg-HMQC fluorescence enhancement complexes together with their associated frontier molecular orbitals HOMO and LUMO

At a working pH of 4–6, the interaction of mercury ions (Hg²⁺) with the designed sensor was thoroughly investigated and the coordination of mercury induces a significant fluorescence quenching, which is consistent with the 1:2 binding stoichiometry observed. The coordinating functional groups are predominantly in their neutral protonation states, as supported by UV-Vis studies. Under these mildly acidic conditions, the sensor exhibits optimal binding behavior and fluorescence response, indicating that the neutral forms of the donor atoms—the oxygen atoms of the hydroxyl and carboxyl functional groups—play a crucial role in complex formation. Considering these information, DFT calculation employing the method explain above was conducted and the obtained chemical geometry support the formation of a tetracoordinated Hg²⁺ complex, Figure 12A. At pH 7 and above, experimental findings indicate that the coordination between HMQC and Hg²⁺ adopts a 1:1 stoichiometry, in which the ligating oxygen atoms are deprotonated. To gain structural insight into this complex, DFT calculations were performed, and the optimized geometry is presented in Fig. 12B. Notably, the coordination to Hg²⁺ in this complex is significantly stronger due to the presence of negatively charged donor atoms from the deprotonated sensor. Compared to the 1:2 stoichiometry observed at lower pH, the 1:1 complex formed at neutral pH exhibits not only greater structural rigidity but also enhanced orbital delocalization and reduced non-radiative decay pathways. These combined effects account for the significant fluorescence enhancement observed upon Hg²⁺ coordination. This enhanced structural rigidity may reduce the conformational flexibility of the sensor, thereby suppressing non-radiative decay pathways and contributing to the observed fluorescence enhancement.

Our computational investigation was further expanded to visualize the electronic distribution of the complex through the Highest Occupied Molecular Orbital (HOMO) and Lowest Unoccupied Molecular Orbital (LUMO). Notably, the HOMO is mainly localized on the aromatic rings, with negligible contribution from the coordinating oxygen atoms, indicating that these atoms are more involved in binding rather than electron donation. Meanwhile, the electron delocalization in the 1:1 Hg–HMQC complex (Fig. 12B) exhibits notable contributions from the deprotonated, negatively charged oxygen atoms involved in coordination. This redistribution results in an expanded delocalization of electron density across the entire sensor framework, compared to the earlier, less coordinated state. Such enhanced conjugation and electronic coupling may further contribute to the observed fluorescence enhancement by stabilizing the excited state and reducing non-radiative decay pathways. Conversely, the LUMO over the quenching complex displays pronounced electron deficiency in the coordination region, highlighting the electron-accepting nature of the mercury binding site. This redistribution of electron density in the complex plays a crucial role in the observed fluorescence quenching observed. In particular, the localization of electron deficiency around the metal-binding site is likely responsible for non-radiative decay processes, which effectively suppress the excited-state fluorescence. Compared to the higher-order complex, the 1:1 coordination structure displays a modified LUMO profile, where a larger portion of the sensor’s framework contributes to electron delocalization. This broader distribution may facilitate more efficient excited-state stabilization, which could be associated with the observed enhancement in fluorescence. The alignment of stoichiometry, spectroscopic response, and electronic structure analysis strongly supports the proposed binding model and provides insight into the sensing mechanism. It is important to note that in solution, the coordination sphere of Hg²⁺ may involve additional ligands, including water molecules or counteranions such as NO₃⁻. This is particularly relevant at neutral or slightly basic pH, where hydroxide ions or deprotonated ligand sites may compete for coordination. These factors could contribute to forming a more complex coordination geometry than the simplified model suggests. Future theoretical studies will include explicit solvent and ion models to more accurately simulate realistic solution-phase behavior.

### Mechanistic Interpretation

**Scheme 2:**
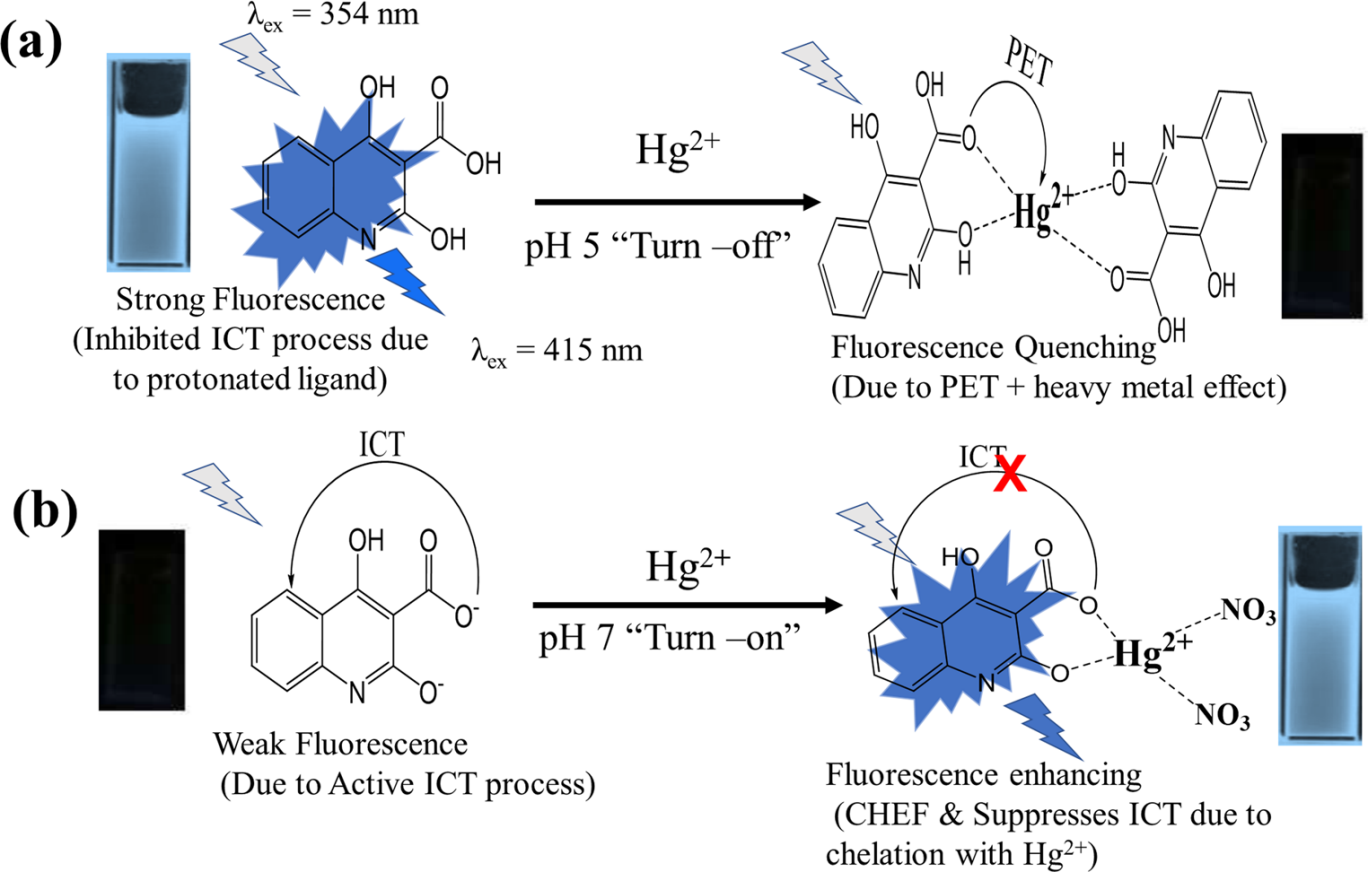
Schematic presentation of coordination of HMQC with Hg^2+^ ions at (**a**) pH 5 and (**b**) pH 7

The fluorescence response of the HMQC chemosensor is strongly pH-dependent in both the absence and presence of Hg²⁺, and this dual behavior can be rationalized by considering the protonation state of the ligand, the nature of its metal coordination, and the resulting electronic structure, as illustrated in Scheme 2.

At acidic pH (4–6), the hydroxyl (–OH) and carboxyl (–COOH) groups of HMQC are predominantly protonated, preserving strong π-conjugation across the quinoline core while minimizing the electron-donating ability of the peripheral sites. In this form, intramolecular charge transfer (ICT) is effectively suppressed, and radiative decay is favored, leading to high intrinsic fluorescence of the free probe. Upon addition of Hg²⁺ at pH 5, Job’s plot analysis and DFT calculations (Fig. 12A) indicate the formation of a relatively flexible 1:2 (Hg: HMQC) complex. This weak and dynamic coordination activates non-radiative decay channels, most likely through photoinduced electron transfer (PET) and increased conformational freedom, which collectively result in efficient quenching of the excited state. The apparent stability constant obtained from Stern–Volmer analysis at this pH (Kₐ = 1.30 × 10⁵ L·mol⁻¹, Sect. 3.8) is consistent with a moderately stable, PET-active 1:2 complex that lacks a rigid chelated geometry. In contrast, at neutral pH (~ 7), partial deprotonation of the –COOH and –OH groups generates –COO⁻ and –O⁻ anions, which are significantly stronger electron donors. This enhances ICT from the peripheral donor sites toward the electron-deficient quinoline moiety, stabilizing a non-emissive, charge-separated excited state and strongly quenching the fluorescence of the free ligand. The introduction of Hg²⁺ under these conditions reverses this situation. Job’s plots and fluorescence titration data indicate the formation of a 1:1 (Hg: HMQC) complex, while DFT-optimized geometries (Fig. 12B) reveal a rigid chelate with extensive HOMO–LUMO delocalization and pronounced metal–ligand orbital overlap. Chelation under these conditions sequesters the lone pairs responsible for ICT, increases molecular rigidity, and restores radiative decay through a classical Chelation-Enhanced Fluorescence (CHEF) effect, producing a strong turn-on response. The higher stability constant derived from the Benesi–Hildebrand treatment at pH 7 (Kₐ = 1.12 × 10⁶ L·mol⁻¹, Sect. 3.8) reflects the much stronger affinity of Hg²⁺ for the deprotonated ligand and explains why the fluorescence enhancement reaches saturation at very low Hg²⁺ concentrations (~ 50 nM), despite the higher bulk concentration of HMQC. Importantly, the Benesi–Hildebrand plot (Fig. 1S) exhibits excellent linearity, which is characteristic of a single-site 1:1 binding model; such linear relationships would not be observed for higher-order stoichiometries (e.g., 1:2 or 2:1). This strong linear behavior therefore provides additional quantitative confirmation that the emissive complex formed at neutral pH follows a true 1:1 binding mode.

The plateau in emission thus corresponds to saturation of a single high-affinity emissive binding site per HMQC molecule rather than stoichiometric equivalence on a bulk concentration basis. Although the experimentally observed stoichiometry at neutral pH is 1:1, Hg²⁺ is expected to achieve its preferred four-coordinate environment through additional interactions with solvent molecules or counteranions such as NO₃⁻ originating from Hg(NO₃)₂. Such auxiliary coordination completes the metal’s coordination sphere without altering the spectroscopically observed 1:1 ligand–metal binding model. The pH-dependent coordination behavior can be further rationalized using Pearson’s Hard and Soft Acids and Bases (HSAB) theory in conjunction with computational and experimental data [51–53]. While Hg²⁺ is a soft Lewis acid and oxygen donors are traditionally considered hard bases, deprotonation at pH 7 and embedding of the oxygen atoms within an extended π-conjugated framework lead to resonance stabilization and partial delocalization of charge, effectively softening the donor sites. This borderline-soft character makes the deprotonated HMQC particularly compatible with the soft-acidic Hg²⁺ center, favoring the formation of a strongly bound, rigid 1:1 chelate. At lower pH, the fully protonated oxygen donors behave more like classical hard bases, resulting in weaker and more flexible 1:2 coordination that promotes non-radiative quenching rather than CHEF [45, 55, 56].

The experimental selectivity profile (Sect. 3.11, Fig. 11) further supports this mechanistic picture. When HMQC was tested against a panel of metal ions (Fe³⁺, Cu²⁺, Zn²⁺, Ni²⁺, Pb²⁺, Co²⁺, Mg²⁺, Na⁺, and Cd²⁺), none of these cations produced a comparable fluorescence turn-on response, despite being present at the same concentration (50 nM). These ions either fail to achieve the same degree of coordination strength and orbital alignment, due to HSAB mismatch (hard–hard or borderline–hard interactions), or do not stabilize the rigid emissive geometry observed for the Hg²⁺ complex. The clear difference between the stability constants at pH 5 and pH 7, the distinct DFT-predicted electronic structures of the 1:2 and 1:1 complexes, and the unique fluorescence enhancement induced exclusively by Hg²⁺ together establish a coherent, pH-controlled dual-mode sensing mechanism. At low pH, HMQC is intrinsically emissive but is quenched upon 1:2 binding with Hg²⁺, whereas at neutral pH, the ligand is intrinsically non-emissive due to ICT but becomes highly fluorescent upon formation of the 1:1 chelate. This integrated mechanistic framework unifies spectroscopic, computational, and binding data into a predictive model for highly selective and sensitive Hg²⁺ detection in aqueous environments.

### Analytical Application

The practical applicability of HMQC as a chemosensor for detecting Hg²⁺ in real-world water samples was evaluated by analyzing spiked water samples collected from Ismailia city, Egypt. The water samples were pretreated by filtration through a microfiltration membrane to remove suspended particles, followed by pH adjustment to 7.4 to ensure optimal conditions for the probe’s fluorescence response. A series of Hg²⁺ solutions with concentrations of 10, 20, 30, 40, and 50 nM were spiked into the water samples, with HMQC (100.0 µM) used as the chemosensor. Fluorescence intensities were recorded at 415 nm for each sample, and the measurements were repeated three times to ensure accuracy and reproducibility. The concentrations of Hg²⁺ in the spiked samples were calculated using the calibration curve obtained from the probe’s fluorescence response. The results, summarized in Table 3, demonstrate the accuracy, precision, and robustness of HMQC for Hg²⁺ detection in real-world samples. The recovery rates ranged from 92% to 108%, indicating excellent agreement between the spiked and measured Hg²⁺ concentrations. Additionally, the relative standard deviation (RSD) values were consistently below 5%, confirming the reproducibility of the measurements. These metrics highlight the reliability of HMQC for practical applications in environmental monitoring. The probe exhibited consistent fluorescence responses across all tested concentrations, with higher Hg²⁺ concentrations resulting in a proportional increase in fluorescence intensity. For example, in Sample 1, recovery rates ranged from 92% to 103%, with RSD values between 1.92% and 4.57%. Similar performance was observed for the other samples, with minimal variability. Slightly higher recovery values, such as 108% at 30 nM in Sample 4, may be attributed to minor matrix effects or slight deviations during fluorescence measurements. The use of pretreated water samples and controlled experimental conditions minimized potential interferences, allowing HMQC to function effectively even in complex matrices. The adjustment of pH to 7.4 ensured that the probe operated under optimal conditions, where fluorescence enhancement upon Hg²⁺ binding is maximized. This consistency across various samples and concentrations demonstrates the versatility and adaptability of HMQC for detecting Hg²⁺ in real-world scenarios. These findings validate the practical utility of HMQC as a selective, sensitive, and reliable chemosensor for Hg²⁺ detection in environmental water samples. The probe’s high recovery rates, low RSD values, and ability to function effectively in complex matrices underscore its potential for real-world applications in environmental monitoring and water quality assessment.

**Table 3:** Detection of Hg^2+^ in real-world water samples using HMQC

Samples	Hg^2+^ spiked	Hg^2+^ found Mean ^a^ ± SD ^b^	Recovery	RSD%
Sample 1	10	10.32 ± 0.35	103%	2.51
	20	20.59 ± 0.54	103%	4.57
	30	27.57 ± 0.52	92%	3.62
	40	39.25 ± 0.74	98%	3.87
	50	49.57 ± 0.45	99%	1.92
Sample 2	10	9.87 ± 0.25	99%	4.32
	20	19.45 ± 0.42	97%	3.85
	30	29.54 ± 0.44	98%	2.77
	40	40.35 ± 0.055	101%	1.45
	50	48.23 ± 0.35	96%	1.02
Sample 3	10	10.55 ± 0.54	106%	3.33
	20	20.45 ± 0.54	102%	2.78
	30	28.23 ± 0.87	94%	4.65
	40	41.22 ± 0.65	103%	1.04
	50	49.78 ± 0.47	100%	4.32
Sample 4	10	9.83 ± 0.22	98%	2.36
	20	19.78 ± 0.35	99%	2.54
	30	32.52 ± 0.98	108%	2.66
	40	37.56 ± 0.78	94%	1.06
	50	47.65 ± 0.42	95%	2.35

a Mean of three measurements

b Standard deviation

## Conclusion

This study presents the successful development and comprehensive evaluation of a novel quinoline-based chemosensor, HMQC, for the detection of mercury ions (Hg²⁺) in aqueous environments. The dual fluorescence behavior of HMQC, exhibiting quenching at acidic pH (pH 5) and enhancement at neutral pH (pH 7), provides a versatile platform for both quantitative and qualitative analysis. The probe demonstrated high selectivity for Hg²⁺ over other competing metal ions, with significant fluorescence changes even in complex matrices, emphasizing its potential for real-world applications. Key findings include the superior performance of HMQC at pH 7, where it achieved a lower limit of detection (LOD) of 1.47 nM and a broader linear range (4.84–50 nM), making it suitable for trace-level mercury detection in compliance with international water quality standards, such as those set by the World Health Organization (WHO) and the European Union (EU). The results of the reversibility study further highlighted the robustness and reusability of the probe, with consistent fluorescence responses over multiple binding and displacement cycles using EDTA. Despite the promising results, some limitations were observed. The sensitivity of the probe, while sufficient for regulatory compliance, could be further enhanced for ultra-trace level detection in highly sensitive applications. Additionally, the pH-dependent behavior, while versatile, requires careful optimization of the sample environment to achieve the best analytical performance. The exceptional selectivity of HMQC toward Hg²⁺ arises from a combination of structural, electronic, and theoretical factors. Although the primary donor atoms in HMQC are oxygen-based and traditionally classified as hard bases, their deprotonation at neutral pH within a conjugated aromatic system alters their electronic nature, making them more compatible with soft Lewis acids like Hg²⁺. This alignment, supported by DFT calculations, enables strong and selective 1:1 complexation, leading to a distinct fluorescence “turn-on” via the Chelation-Enhanced Fluorescence (CHEF) mechanism. Comparative experimental studies further confirm that other metal ions do not elicit similar responses under identical conditions, reinforcing the probe’s practical selectivity for mercury sensing in biologically and environmentally relevant media. Future research could explore structural modifications of HMQC to enhance its sensitivity and extend its selectivity to other heavy metal ions. Furthermore, integrating the probe into portable sensing devices or microfluidic platforms could provide a practical solution for on-site and real-time monitoring. The novelty of this work lies in the dual fluorescence response and high selectivity of HMQC, which, combined with its ease of synthesis and robust performance in diverse conditions, makes it a significant advancement in the field of mercury detection. The probe’s adaptability to both acidic and neutral environments and its alignment with stringent water quality standards highlight its potential for widespread environmental and industrial applications. This study sets the foundation for future innovations in chemosensor design and underscores the importance of developing sensitive, selective, and versatile tools for environmental monitoring and public health protection.

## Supplementary Information

Below is the link to the electronic supplementary material.

Supplementary Material 1

## Data Availability

All data generated or analyzed during this study are included in this published article.

## References

[1] Harris HH, Pickering IJ, George GN (2003) The chemical form of Mercury in Fish. Science (1979) 301:1203–1203. 10.1126/science.1085941 10.1126/science.1085941 12947190

[2] Tchounwou PB, Yedjou CG, Patlolla AK, Sutton DJ (2012) Heavy metal toxicity and the environment. pp 133–164 10.1007/978-3-7643-8340-4_6 PMC4144270 22945569

[3] Neathery MW, Miller WJ (1975) Metabolism and toxicity of Cadmium, Mercury, and lead in animals: a review. J Dairy Sci 58:1767–1781. 10.3168/jds.S0022-0302(75)84785-0 1107364 10.3168/jds.S0022-0302(75)84785-0

[4] Jaishankar M, Tseten T, Anbalagan N et al (2014) Toxicity, mechanism and health effects of some heavy metals. Interdiscip Toxicol 7:60–72. 10.2478/intox-2014-0009 26109881 10.2478/intox-2014-0009 PMC4427717

[5] Udhayakumari D (2022) Review on fluorescent sensors-based environmentally related toxic mercury ion detection. J Incl Phenom Macrocycl Chem 102:451–476. 10.1007/s10847-022-01138-1

[6] Liu Y, Su X, Liu H et al (2025) Construction of eco-friendly dual carbon dots ratiometric fluorescence probe for highly selective and efficient sensing mercury ion. J Environ Sci 148:1–12. 10.1016/j.jes.2024.01.013 10.1016/j.jes.2024.01.013 39095148

[7] Leng G, Feng L, Li S-B et al (2013) Determination of mercury (Hg) in sediment by a sequential injection (SI) system with cold vapor generation atomic fluorescence spectrometry (CVAFS) detection after a rapid and mild Microwave-Assisted digestion. Environ Forensics 14:9–15. 10.1080/15275922.2012.729003

[8] Hofer I, Gremaud M, Marchese A, Le Bouhellec S (2017) Determination of mercury in aerosol by inductively coupled plasma mass spectrometry. Beiträge Zur Tabakforschung International/Contributions Tob Res 27:186–194. 10.1515/cttr-2017-0020

[9] Anderson KA (2000) Mercury analysis in environmental samples by cold vapor Techniques. In: encyclopedia of analytical chemistry. Wiley

[10] Aliberti A, Vaiano P, Caporale A et al (2017) Fluorescent chemosensors for Hg2 + detection in aqueous environment. Sens Actuators B Chem 247:727–735. 10.1016/j.snb.2017.03.026

[11] Gholami MD, Manzhos S, Sonar P et al (2019) Dual chemosensor for the rapid detection of mercury(< scp > ii) pollution and biothiols. Analyst 144:4908–4916. 10.1039/C9AN01055F 31312834 10.1039/c9an01055f

[12] Zavalishin MN, Gamov GA (2025) Fluorescein acetone hydrazone: a highly sensitive probe for Hg2 + ion detection and computational insights. J Mol Struct 1348. 10.1016/j.molstruc.2025.143405

[13] Zavalishin MN, Pogonin AE, Gamov GA (2025) Hg2+-induced hydrolysis of fluorescein hydrazone: a new fluorescence probe for selective recognition Hg2 + in an aqueous solution. J Mol Struct 1334. 10.1016/j.molstruc.2025.141930

[14] Zavalishin MN, Kiselev AN, Isagulieva AK et al (2024) Shedding light on heavy metal contamination: Fluorescein-based chemosensor for selective detection of Hg2 + in water. Int J Mol Sci 25. 10.3390/ijms25063186 10.3390/ijms25063186 PMC10970617 38542159

[15] Alshareef FM, Algethami JS, Alhamami MAM et al (2024) Recent advancement in organic fluorescent and colorimetric chemosensors for the detection of Al3 + ions: A review (2019–2024). J Environ Chem Eng 12

[16] Ullah I, Muhammad M, Khan S et al (2025) Indole-based Hydrazide as optical chemosensor for the determination of Cu 2 + and Zn 2+. Int J Environ Anal Chem 1–14. 10.1080/03067319.2025.2511978

[17] Khan S, Muhammad M, Alosaimi EH et al (2025) Ultrasensitive fluorimetric detection of Hg2 + Using a Thiourea-Based chemosensor. J Fluoresc. 10.1007/s10895-025-04193-2 40000550 10.1007/s10895-025-04193-2

[18] Muhammad M, Khan S, Al-Saidi HM et al (2025) Novel Thiourea-based chemosensor for ultrasensitive and selective detection of Hg2+. J Fluoresc. 10.1007/s10895-025-04147-8 39918672 10.1007/s10895-025-04147-8

[19] Alhamami MAM, Algethami JS, Khan S (2024) A review on thiazole based colorimetric and fluorimetric chemosensors for the detection of heavy metal ions. Crit Rev Anal Chem 54:2689–2713 37029905 10.1080/10408347.2023.2197073

[20] Gul Z, Khan S, Khan E (2024) Organic molecules containing N, S and O heteroatoms as sensors for the detection of Hg(II) Ion; coordination and efficiency toward detection. Crit Rev Anal Chem 54:1525–1546 36122189 10.1080/10408347.2022.2121600

[21] Algethami S, Al-Saidi J, Alosaimi HM EH, et al (2025) Recent advancements in fluorometric and colorimetric detection of Cd2 + Using organic chemosensors: A review (2019–2024). Crit Rev Anal Chem 55:1165–1184 38655923 10.1080/10408347.2024.2339968

[22] Mariammal M, Sahane N, Tiwari S (2023) Water-soluble anionic N-confused porphyrin for sensitive and selective detection of heavy metal pollutants in aqueous environment. Anal Sci 39:1317–1325. 10.1007/s44211-023-00341-5 37140885 10.1007/s44211-023-00341-5

[23] Ke X, Fan Y, Zhou J, Huang Z (2020) A novel coumarin-derived dithioacetal chemosensor for trace detection of hg 2 + in real water samples. J Chem Res 44:142–147. 10.1177/1747519819890561

[24] Liu Y, Yang E-B, Han R et al (2014) A new rhodamine-based fluorescent chemosensor for mercury in aqueous media. Chin Chem Lett 25:1065–1068. 10.1016/j.cclet.2014.04.033

[25] El-Shekheby HA, Mangood AH, Hamza SM et al (2014) A highly efficient and selective turn‐on fluorescent sensor for hg 2+, ag + and ag nanoparticles based on a coumarin dithioate derivative. Luminescence 29:158–167. 10.1002/bio.2521 23703858 10.1002/bio.2521

[26] He X, Zhang J, Liu X et al (2014) A novel BODIPY-based colorimetric and fluorometric dual-mode chemosensor for Hg2 + and Cu2+. Sens Actuators B Chem 192:29–35. 10.1016/j.snb.2013.10.093

[27] Kumari N, Singh S, Baral M, Kanungo BK (2023) Schiff bases: a versatile fluorescence probe in sensing cations. J Fluoresc 33:859–893. 10.1007/s10895-022-03135-6 36633727 10.1007/s10895-022-03135-6

[28] Wu X, Yang W, Liu M et al (2011) Vapor generation in dielectric barrier discharge for sensitive detection of mercury by inductively coupled plasma optical emission spectrometry. J Anal Spectrom 26:1204. 10.1039/c1ja10016e

[29] Peng L, Guo H, Wu N et al (2024) A dual-functional fluorescence probe CDs@ZIF-90 for highly specific detection of Al3 + and Hg2 + in environmental water samples. Anal Chim Acta 1288:342171. 10.1016/j.aca.2023.342171 38220302 10.1016/j.aca.2023.342171

[30] Li C, Xu R, Liu X et al (2024) Visible self-luminous indium-based metal–organic framework for electrochemiluminescence detection of Hg2+. Sens Actuators B Chem 405:135383. 10.1016/j.snb.2024.135383

[31] Khairy GM, Goda RM, Anwar ZM et al (2024) Luminescent and time-resolved determination of Gemifloxacin mesylate in pharmaceutical formulations and spiked blood plasma samples using a lanthanide complex as a probe. Anal Methods 16:2556–2568. 10.1039/D4AY00236A 38592494 10.1039/d4ay00236a

[32] Khairy GM, Ragab SM, Moawed EA et al (2024) Uncovering an effecient binary system as a chemosensor for visual and fluorescence detection of chromium (VI) in water samples. Spectrochim Acta Mol Biomol Spectrosc 321:124729. 10.1016/j.saa.2024.124729 10.1016/j.saa.2024.124729 38955073

[33] Elbayoumy E, Elhendawy M, Gaafar MM et al (2024) Novel fluorescent sensor based on triazole-pyridine derivative for selective detection of mercury (II) ions in different real water samples: experimental and DFT calculations. J Mol Liq 401:124589. 10.1016/j.molliq.2024.124589

[34] Khan J (2024) Synthesis and applications of fluorescent chemosensors: a review. J Fluoresc 34:2485–2494. 10.1007/s10895-023-03455-1 37906359 10.1007/s10895-023-03455-1

[35] Formica M, Fusi V, Giorgi L, Micheloni M (2012) New fluorescent chemosensors for metal ions in solution. Coord Chem Rev 256:170–192. 10.1016/j.ccr.2011.09.010

[36] Uglov AN, Bessmertnykh-Lemeune A, Guilard R et al (2014) Optical methods for the detection of heavy metal ions. Rus Chem Rev 83:196–224. 10.1070/RC2014v083n03ABEH004414

[37] Wu H, Wang S, Ding J et al (2020) Effect of π-conjugation on solid-state fluorescence in highly planar dyes bearing an intramolecular H-bond. Dyes Pigm 182:108665. 10.1016/j.dyepig.2020.108665

[38] Sushma SS, Ghosh KS (2024) Fluorescence chemosensing and bioimaging of metal ions using schiff base probes working through photo-induced electron transfer (PET). Crit Rev Anal Chem 1–32. 10.1080/10408347.2024.2418327 10.1080/10408347.2024.2418327 39559829

[39] Qiao T, Shi W, Zhuang H et al (2024) Fluorescence enhancement mechanism of new 4-Cyanobiphenyl-based schiff base probe by coordination interaction with cadmium ion. J Lumin 269:120413. 10.1016/j.jlumin.2023.120413

[40] Elmorsi TM, Aysha TS, Machalický O et al (2017) A dual functional colorimetric and fluorescence chemosensor based on benzo[f]fluorescein dye derivatives for copper ions and pH; kinetics and thermodynamic study. Sens Actuators B Chem 253:437–450. 10.1016/j.snb.2017.06.084

[41] Kournoutas F, Kalis IK, Fecková M et al (2020) The effect of protonation on the excited state dynamics of pyrimidine chromophores. J Photochem Photobiol Chem 391:112398. 10.1016/j.jphotochem.2020.112398

[42] Roy A, Nandi M, Roy P (2021) Dual chemosensors for metal ions: a comprehensive review. TRAC Trends Anal Chem 138:116204. 10.1016/j.trac.2021.116204

[43] Chen S-H, Jiang K, Liang Y-H et al (2023) Fine-tuning benzazole-based probe for the ultrasensitive detection of Hg2 + in water samples and seaweed samples. Food Chem 428:136800. 10.1016/j.foodchem.2023.136800 37433252 10.1016/j.foodchem.2023.136800

[44] Packirisamy V, Ayyadurai B, Perumal D et al (2023) Green synthesis of R-phycoerythrin protected gold nanoclusters for sensitive detection of mercury(II) ions and their antibacterial properties. J Mater Res 38:3009–3021. 10.1557/s43578-023-01021-w

[45] Valeur B, Berberan-Santos MN (2012) Molecular fluorescence. Wiley

[46] Wu D, Sedgwick AC, Gunnlaugsson T et al (2017) Fluorescent chemosensors: the past, present and future. Chem Soc Rev 46:7105–7123. 10.1039/C7CS00240H 29019488 10.1039/c7cs00240h

[47] Kim HN, Ren WX, Kim JS, Yoon J (2012) Fluorescent and colorimetric sensors for detection of lead, cadmium, and mercury ions. Chem Soc Rev 41:3210–3244. 10.1039/C1CS15245A 22184584 10.1039/c1cs15245a

[48] Lakowicz JR (2006) Principles of fluorescence spectroscopy. Springer US, Boston, MA

[49] Ding H, Zheng C, Li B et al (2016) A rhodamine-based sensor for hg 2 + and resultant complex as a fluorescence sensor for I –. RSC Adv 6:80723–80728. 10.1039/C6RA17861H

[50] Wang M, Wen J, Qin Z, Wang H (2015) A new coumarin–rhodamine FRET system as an efficient ratiometric fluorescent probe for Hg2 + in aqueous solution and in living cells. Dyes Pigm 120:208–212. 10.1016/j.dyepig.2015.04.013

[51] Ge Y, Liu A, Ji R et al (2017) Detection of Hg2 + by a FRET ratiometric fluorescent probe based on a novel pyrido[1,2-a]benzimidazole-rhodamine system. Sens Actuators B Chem 251:410–415. 10.1016/j.snb.2017.05.097

[52] Dong M, Tang J, Lv Y et al (2020) A dual–function fluorescent probe for hg (II) and Cu (II) ions with two mutually independent sensing pathways and its logic gate behavior. Spectrochim Acta Mol Biomol Spectrosc 226:117645. 10.1016/j.saa.2019.117645 10.1016/j.saa.2019.117645 31622829

[53] Yang Z, Chen S, Zhao Y et al (2018) Hg2 + chromogenic and fluorescence indicators based on Rhodamine derivatives bearing thiophene group. Sens Actuators B Chem 266:422–428. 10.1016/j.snb.2018.03.148

[54] Gao Y, Zhang C, Peng S, Chen H (2017) A fluorescent and colorimetric probe enables simultaneous differential detection of Hg2 + and Cu2 + by two different mechanisms. Sens Actuators B Chem 238:455–461. 10.1016/j.snb.2016.07.076

[55] Pearson RG (1963) Hard and soft acids and bases. J Am Chem Soc 85:3533–3539. 10.1021/ja00905a001

[56] Yuan L, Lin W, Zheng K et al (2013) Far-red to near infrared analyte-responsive fluorescent probes based on organic fluorophore platforms for fluorescence imaging. Chem Soc Rev 42:622–661. 10.1039/C2CS35313J 23093107 10.1039/c2cs35313j

