# Supplementary material for: Novel Quinoline Chemosensor with Dual-Mode Fluorescence and DFT-Backed Mechanism for Mercury(II) Sensing
Source: J Fluoresc. 2026 Feb 11;36(3):2571–89. doi: 10.1007/s10895-025-04702-3 (PMC12992395; doi:10.1007/s10895-025-04702-3)
Supplement: Supplementary file 1 — Supplementary Material 1 [file 10895_2025_4702_MOESM1_ESM.docx]

Supporting Information

**Novel Quinoline Chemosensor with Dual-Mode Fluorescence and DFT-Backed Mechanism for Mercury(II) Sensing**

Gasser M. Khairy ^1*^; Bader M. Alanazi ^2^; Yasser A Attia ^3^; Mohamed M. Aboelnga,^4,5^ Randa M. Abdel Hameed ^2^

*^1^ Chemistry Department, Faculty of Science, Suez Canal University, 41522 Ismailia, Egypt*

*^2^ Department of chemistry, Faculty of Science Cairo University 12613 Giza Egypt*

*^3^ National Institute of Laser Enhanced Sciences Cairo University 12613 Giza Egypt*

*^4^ Chemistry Department, Faculty of Science, Damietta University, New Damietta 34517, Egypt*

*^5^ King Salman International University, Faculty of Basic Sciences, Ras Sudr, 46612, South Sinai, Egypt*

Figure 1S. Benesi–Hildebrand plots for 1:1 complex interaction of Hg^2+^ with

HMCQ.
